# Quantitative Proteomic Analysis of Cyanide and Mercury Detoxification by Pseudomonas pseudoalcaligenes CECT 5344

**DOI:** 10.1128/spectrum.00553-23

**Published:** 2023-07-11

**Authors:** Karolina A. Biełło, Alfonso Olaya-Abril, Purificación Cabello, Gema Rodríguez-Caballero, Lara P. Sáez, Conrado Moreno-Vivián, Víctor Manuel Luque-Almagro, María Dolores Roldán

**Affiliations:** a Departamento de Bioquímica y Biología Molecular, Edificio Severo Ochoa, Campus de Rabanales, Universidad de Córdoba, Córdoba, Spain; b Departamento de Botánica, Ecología y Fisiología Vegetal, Edificio Celestino Mutis, Campus de Rabanales, Universidad de Córdoba, Córdoba, Spain; Universita degli Studi Roma Tre Dipartimento di Scienze

**Keywords:** arsenic, biodegradation, cyanide, heavy metals, mercury, *Pseudomonas*

## Abstract

The cyanide-degrading bacterium Pseudomonas pseudoalcaligenes CECT 5344 uses cyanide and different metal-cyanide complexes as the sole nitrogen source. Under cyanotrophic conditions, this strain was able to grow with up to 100 μM mercury, which was accumulated intracellularly. A quantitative proteomic analysis by liquid chromatography-tandem mass spectrometry (LC-MS/MS) has been applied to unravel the molecular basis of the detoxification of both cyanide and mercury by the strain CECT 5344, highlighting the relevance of the cyanide-insensitive alternative oxidase CioAB and the nitrilase NitC in the tolerance and assimilation of cyanide, independently of the presence or absence of mercury. Proteins overrepresented in the presence of cyanide and mercury included mercury transporters, mercuric reductase MerA, transcriptional regulator MerD, arsenate reductase and arsenical resistance proteins, thioredoxin reductase, glutathione *S*-transferase, proteins related to aliphatic sulfonates metabolism and sulfate transport, hemin import transporter, and phosphate starvation induced protein PhoH, among others. A transcriptional study revealed that from the six putative *merR* genes present in the genome of the strain CECT 5344 that could be involved in the regulation of mercury resistance/detoxification, only the *merR2* gene was significantly induced by mercury under cyanotrophic conditions. A bioinformatic analysis allowed the identification of putative MerR2 binding sites in the promoter regions of the regulatory genes *merR5*, *merR6*, *arsR*, and *phoR*, and also upstream from the structural genes encoding glutathione *S*-transferase (*fosA* and *yghU*), dithiol oxidoreductase (*dsbA*), metal resistance chaperone (*cpxP*), and amino acid/peptide extruder involved in quorum sensing (*virD*), among others.

**IMPORTANCE** Cyanide, mercury, and arsenic are considered very toxic chemicals that are present in nature as cocontaminants in the liquid residues generated by different industrial activities like mining. Considering the huge amounts of toxic cyanide- and mercury-containing wastes generated at a large scale and the high biotechnological potential of *P. pseudoalcaligenes* CECT 5344 in the detoxification of cyanide present in these industrial wastes, in this work, proteomic, transcriptional, and bioinformatic approaches were used to characterize the molecular response of this bacterium to cyanide and mercury, highlighting the mechanisms involved in the simultaneous detoxification of both compounds. The results generated could be applied for developing bioremediation strategies to detoxify wastes cocontaminated with cyanide, mercury, and arsenic, such as those generated at a large scale in the mining industry.

## INTRODUCTION

Mercury is an environmental hazard that is accumulated in nature at low concentrations as both inorganic and organic forms, such as elemental/metallic (Hg^0^) or bivalent (Hg^II^) mercury, and organomercurials (R-Hg). In addition to natural processes, anthropogenic activities produce up to 7,000 metric tons of mercury per year, which may be toxic disposals in the absence of appropriate waste management strategies (1, 2). Among these human activities include fossil fuel combustion, especially coal; metal ore mining; mercury-containing pesticides used in agriculture; and mercury-based catalysts applied to industrial purposes (1–4). The largest amounts of mercury released to the environment correspond to mining activities, mainly related to gold extraction. In the recovery of precious metals from ores, elevated concentrations of cyanide are also used in the so-called cyanidation process (cyanide leaching), and other pollutants like arsenic, lead, copper, or zinc are usually present as cocontaminants of the cyanide-containing wastes generated from mining industries (5–8). About one million tons/year of these wastewaters from mines are produced, which are stored in artificial ponds that are prone to leaching or dam breaks, posing a major threat to the environment and human health (8).

Elemental mercury is dangerous because of its volatility, and pollution caused by this form of mercury is not restricted to the specific areas where it is released since it may be easily scattered by the wind. Elemental mercury is chemically oxidized to the bivalent form and deposited in aquatic or terrestrial ecosystems (2, 9, 10). Additionally, mercury can undergo further reactions like reduction or methylation. Methylmercury bioaccumulates and biomagnifies in the food chain (11, 12), and humans may be exposed to methylmercury from contaminated fish when ingested. Likewise, mercury salts like mercury chloride also display great acute toxicity (13). Organomercurials are considered very potent toxins that threaten human health and ecosystems. Methylmercury has a half-life of about 44 days in the human body and constitutes a neurotoxin that accumulates in brain, kidney, and liver. Additionally, methylmercury may pass through the placenta, affecting the fetus (1, 2, 14). Mercury is associated with a broad range of severe diseases, such as Minamata disease, acrodynia, attention deficit disorder, and hyperactivity (15, 16).

Considering that mercury may be present naturally in the environment, many different microorganisms have evolved mechanisms to tolerate and detoxify this toxic metal. Microorganisms play a major role in mercury cycling in nature through reduction, oxidation, and/or bioaccumulation (chelation or biosorption) processes (1, 2, 17). Some bacteria like Escherichia coli, *Bacillus*, or *Streptomyces* display an oxidative mechanism that produces the bivalent form of mercury from elemental mercury through a hydroperoxidase-catalase activity (2, 18). Some dissimilatory sulfate-reducing bacteria and dissimilatory iron-reducing bacteria are able to carry out mercury methylation under anaerobic conditions (19, 20), although it is still controversial if methylation may confer resistance to mercury. On the other hand, extracellular biosorption of mercury has been described in several bacterial strains, occurring mainly through the secretion of negatively charged extracellular polymers (17). However, the most widespread mechanism of resistance triggered by bacteria in response to mercury toxicity is based on the occurrence of the *mer* genes (21). The *mer* operons described up to date are considered narrow spectrum when they confer resistance only to inorganic mercury salts or broad spectrum when they facilitate resistance to both organomercurials and inorganic mercury (21–23).

The alkaliphilic bacterium Pseudomonas pseudoalcaligenes CECT 5344 was isolated from sludges of the Guadalquivir River (Córdoba, Spain) near an industrial area with elevated jewelry activity, in which cyanide is used for gold electroplating. The strain CECT 5344 uses free cyanide, metal-cyanide complexes, and cyano derivatives, including cyanate and 3-cyanoalanine, as the sole nitrogen source under alkaline conditions that prevent cyanhydric acid volatilization (24). *P. pseudoalcaligenes* CECT 5344 harbors in its genome, in addition to the gene clusters involved in cyanide resistance and assimilation, several *mer* and *ars* operons that could confer to this bacterium a wide capability to tolerate and detoxify mercury and arsenic (25).

Considering the large amounts of cyanide- and mercury-containing wastes generated in the mining industry and the high biotechnological potential of *P. pseudoalcaligenes* CECT 5344 in the bioremediation of these industrial wastes (8), in this work, the molecular response to cyanide and mercury of this cyanide-degrading bacterium was characterized through liquid chromatography-tandem mass spectrometry (LC-MS/MS), reverse transcription-quantitative PCR (qRT-PCR), and bioinformatic approaches. The results generated could be useful to develop further bioremediation techniques for the detoxification of wastes cocontaminated with cyanide, mercury, and arsenic.

## RESULTS

### Detoxification of cyanide and mercury by *P. pseudoalcaligenes*.

**FIG 1:**
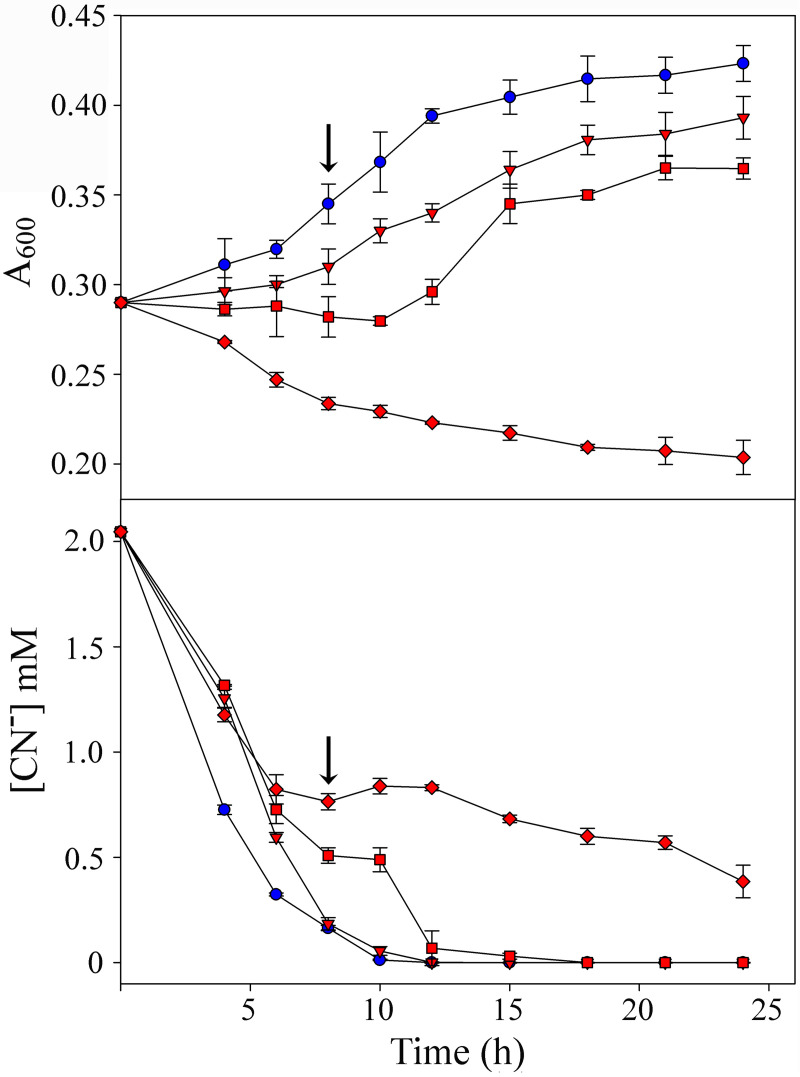
Physiological characterization of *P. pseudoalcaligenes* CECT 5344 cells grown with cyanide as the sole nitrogen source, in the presence or absence of mercury. Growth (top) and cyanide consumption (bottom) of *P. pseudoalcaligenes* CECT 5344 cells in media without mercury (blue circles) or with mercury (red symbols) at 75 μM (triangles), 100 μM (squares), and 150 μM (diamonds). Cells were grown in M9 minimal media with 50 mM sodium acetate and 2 mM sodium cyanide as the carbon and nitrogen source, respectively. CECT 5344 cells for proteomic and qRT-PCR analyses were harvested at 7.5 h of growth (time indicated by the arrow).

To evaluate the ability of the cyanide-degrading bacterium *P. pseudoalcaligenes* CECT 5344 to detoxify cyanide in the presence of mercuric chloride, the MIC and the minimum bactericidal concentration (MBC) were determined in this bacterium. The MIC and MBC of mercury for CECT 5344 cells grown aerobically in M9 minimal media, with 50 mM acetate and 2 mM sodium cyanide as the sole carbon and nitrogen sources, were 200 μM and 300 μM, respectively. In contrast, in the absence of cyanide and with 2 mM ammonium chloride as the nitrogen source, the MIC and MBC of mercury were considerably lower, namely, 10 μM and 17.5 μM, respectively. The growth curves of the CECT 5344 strain were carried out in minimal medium M9 with 2 mM cyanide as the sole nitrogen source in the absence or the presence of different concentrations of mercury, ranging from 75 μM to 150 μM (Fig. 1). The strain CECT 5344 was able to grow with 100 μM mercury, consuming cyanide from the extracellular medium, although this growth was slower than growth with 75 μM mercury. Accordingly, cyanide was totally removed from the medium at 12 h in the presence of 75 μM mercury, but about 18 h were required for the complete consumption of cyanide in the presence of 100 μM mercury (Fig. 1). At higher mercury concentrations, such as 150 μM or above, bacterial growth and cyanide uptake were severely impaired (Fig. 1). Therefore, 75 μM mercury chloride was used to perform further analyses.

Additionally, mercury concentration remaining in the extracellular media or bioaccumulated extracellularly (biosorption) and intracellularly (chelated to biomolecules) was determined by inductively coupled plasma-mass spectrometry (ICP-MS) from CECT 5344 cultures at 7.5 h of growth in media with 2 mM NaCN and 75 μM HgCl_2_, as described in the Materials and Methods. Mercury was neither detected in the extracellular media nor bioaccumulated extracellularly, while the intracellular concentration of mercury was 0.010 ± 0.003 μg/mg protein. In a control experiment carried out without cells, the mercury concentration remained constant throughout the experiment (data not shown).

The analysis of the *P. pseudoalcaligenes* CECT 5344 genome sequence revealed the presence of two *mer* gene clusters in the loci BN5_3800-BN5_3802 (*merP1T1R5*) and BN5_4473-BN5_4479 (*merR6T3P3T5ADE*). These mercury resistance genes code for putative transcriptional regulators MerR and MerD; mercuric transport proteins MerT, MerP, and MerE; and mercuric reductase MerA. In addition, other four *merR* genes (*merR1* to *merR4*), encoding putative MerR transcriptional regulators, were also found scattered throughout the CECT 5344 genome.

### Proteomic analysis of *P. pseudoalcaligenes* grown under cyanotrophic conditions with mercury.

To investigate the global changes in the proteome of *P. pseudoalcaligenes* CECT 5344 provoked by the combination of the two toxic chemicals cyanide and mercury, a proteomic analysis by liquid chromatography-tandem mass spectrometry (LC-MS/MS) was performed in CECT 5344 cells grown under aerobic conditions with 2 mM sodium cyanide as the sole nitrogen source, in the absence (CN) or presence of 75 μM HgCl_2_ (CN + Hg). Cells were harvested at 7.5 h of growth, when they were actively growing, and low concentrations of cyanide remained in the media (Fig. 1). Four independent biological replicates were used in this study, and the principal-component analysis (PCA), clustering of the replicates, and volcano plot were obtained (see Fig. S1 to S3 in the supplemental material). The preliminary qualitative analysis revealed that the overall number of identified proteins (2,689) represented about 60% of the total predicted protein-coding genes (4,435 genes) of the CECT 5344 strain (26).

A further quantitative differential analysis was performed by a comparison of the CN + Hg and CN proteomes (see Table S1 in the supplemental material). In this comparative analysis, the parameter fold change (FC) was used to measure differences in protein expression, which was calculated as the ratio of normalized peptide intensities, CN + Hg/CN. Therefore, proteins shared by both CN + Hg and CN proteomes, but that were nevertheless differentially represented, were considered “overrepresented” in the CN + Hg proteome when the log_2_ FC was ≥1 or “downrepresented” in the CN + Hg proteome when the log_2_ FC was ≤−1. Proteins that were found only in one proteome (either CN + Hg or CN) were considered “exclusive” of that condition. In this quantitative analysis, 21 proteins were exclusive of the CN + Hg proteome and 209 proteins were found differentially expressed (see Fig. S4 in the supplemental material; Table S1). Among these proteins differentially expressed, 45 proteins were overrepresented in the CN + Hg proteome, while 164 proteins were downrepresented in the CN + Hg proteome (Table S1). The gene ontology (GO) analysis (27) using the comparative study between the proteomes CN and CN + Hg revealed insight into functional protein categories that were affected by the presence of mercury under cyanotrophic conditions. Proteins overrepresented in the absence of mercury (or downrepresented with mercury) belonged mainly to the GO category “urea metabolism,” while proteins overrepresented in the presence of mercury (or downrepresented without mercury) belonged to the GO groups “response to metal ion,” “response to toxic substance,” “response to mercury ion,” “detoxification of inorganic compound,” and “oxidoreductase activity, acting on the CH-NH_2_ group of donors,” among others (see Fig. S5 in the supplemental material).

**FIG 2:**
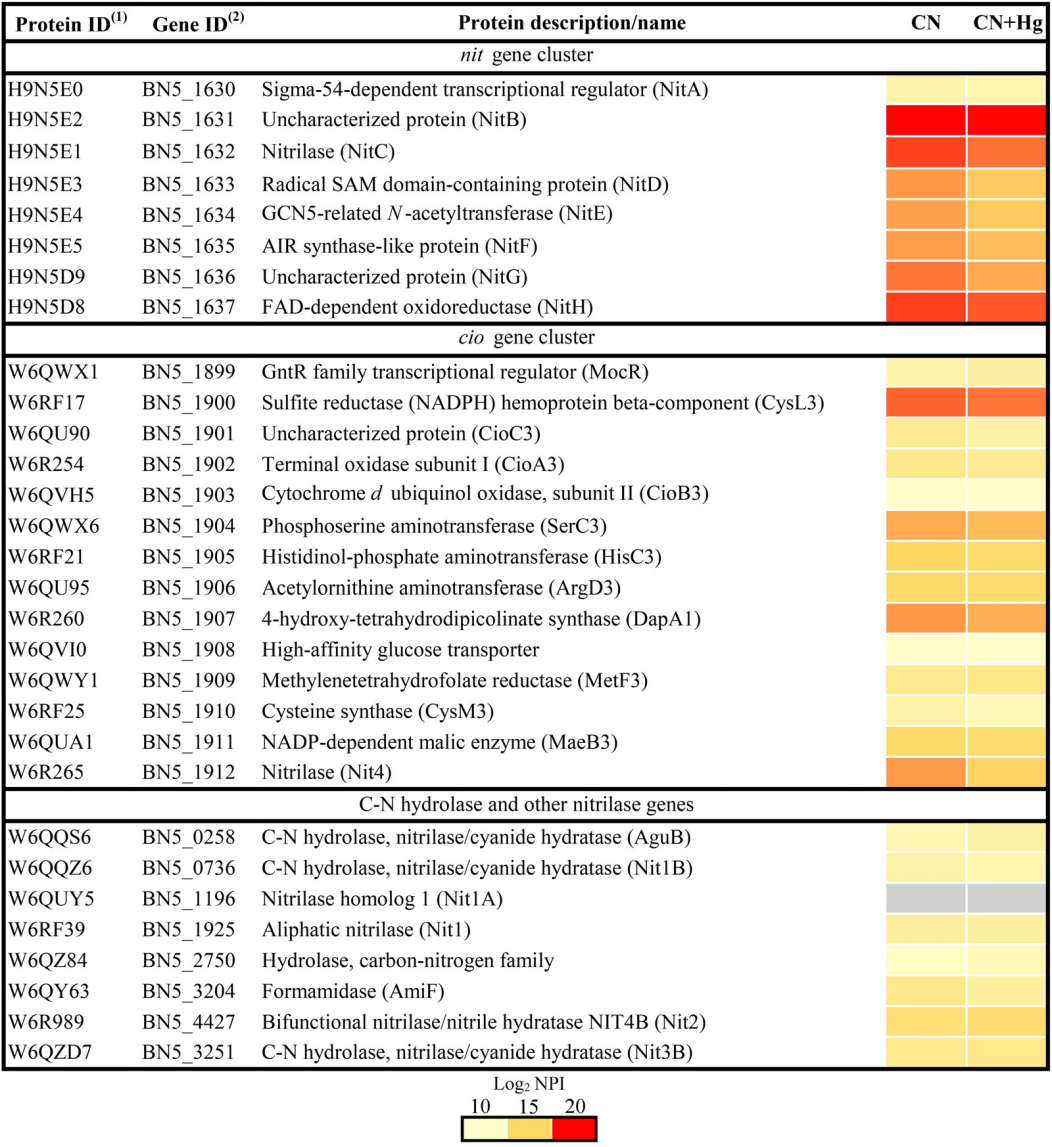
Effect of mercury on proteins involved in cyanide detoxification/assimilation in the *P. pseudoalcaligenes* CECT 5344 strain. Data are shown as a heatmap of the log_2_ of normalized peptide intensity (log_2_ NPI). CN, cyanide-containing media without added mercury; CN + Hg, cyanide-containing media with 75 μM HgCl_2_. (1) Protein code according to the Uniprot database under the accession number UP000032841. (2) Gene annotation from GenBank (accession HG916826.1).

The proteins encoded by the *nit1C* and *cio* gene clusters, which are essential for cyanide assimilation and cyanide-insensitive respiration, respectively (25, 28), were found in both proteomes (CN and CN + Hg) and were not affected significantly by the presence of mercury (Fig. 2).

**FIG 3:**
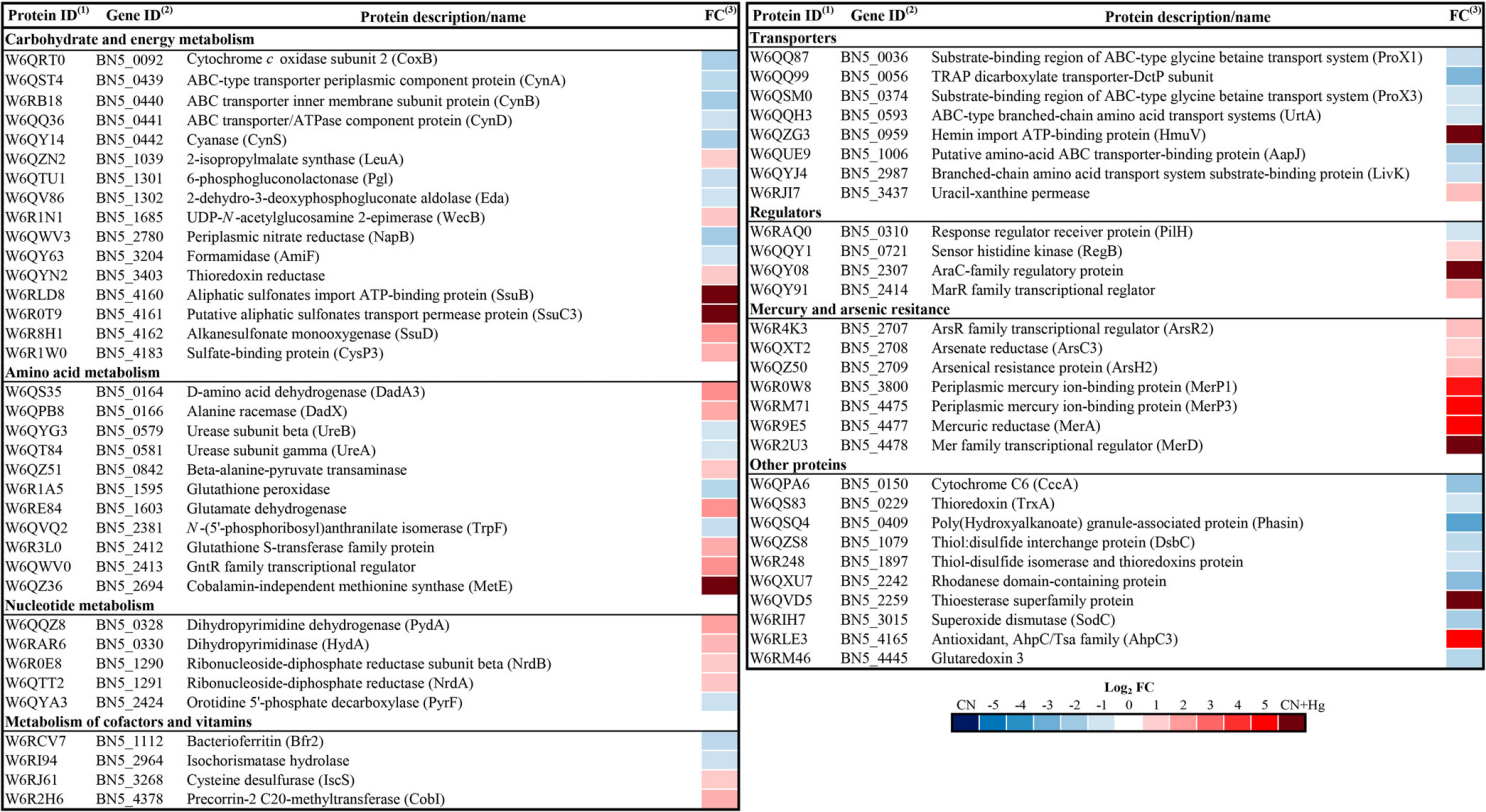
Heatmap of *P. pseudoalcaligenes* CECT 5344 proteins affected by mercury under cyanotrophic conditions. The differential expression of proteins is represented as the log_2_ fold change (FC). The fold change has been calculated as the ratio normalized peptide intensity in CN + Hg/normalized peptide intensity in CN. (1) Protein code according to Uniprot database under the accession number UP000032841. (2) Gene annotation from GenBank (accession HG916826.1).

Some proteins identified in the quantitative analysis were classified functionally. Proteins were grouped into carbohydrate and energy metabolism, nucleotide metabolism, amino acid metabolism, metabolism of cofactors and vitamins, transporters, regulators, mercury and arsenic resistance, and other proteins (Fig. 3). In the first category, proteins involved in sulfur metabolism were highly overrepresented in the CN + Hg proteome, such as the SsuB and SsuC proteins for the transport of aliphatic sulfonates, the alkanesulfonate monooxygenase SsuD, and the sulfate-binding protein CysP3, whereas formamidase and proteins involved in cyanate metabolism were downrepresented in the presence of Hg (Fig. 3; Table S1). Among proteins highly overrepresented in the CN + Hg proteome were Mer proteins for mercury resistance (MerP1, MerP3, MerA, and MerD), arsenical resistance proteins (ArsR2, ArsC3, and ArsH2), proteins involved in the metabolism of amino acids (cobalamin-independent methionine synthase MetE and glutathione *S*-transferase) and nucleotides (dihydropyrimidine dehydrogenase, dihydropyrimidase, and ribonucleotide-diphosphate reductase), the synthesis of cofactors (precorrin-2 C20-methyltransferase CobI), and some transporters (hemin import ATP-binding HmuV) and regulators (RegB, AraC, and GntR family regulators). In contrast, some proteins downrepresented in the CN + Hg proteome were related to polyhydroxyalkanoate production, dicarboxylate transport, and urea metabolism (Fig. 3; Table S1).

Additionally, a quantitative transcriptional analysis by qRT-PCR was applied to study the transcriptional expression of several genes that code for proteins that were found over- or downrepresented in the CN + Hg proteome (Table 1). Genes induced in the presence of mercury were those coding for phosphate starvation-induced protein PhoH, precorrin-2 C20-methyltransferase CobI, alkanesulfonate monooxygenase SsuD, alkyl hydroperoxide reductase AhpC, thioredoxin reductase, transcriptional regulator ArsR2, arsenate reductase ArsC3, glutathione *S*-transferase, and ribonucleoside-diphosphate reductase NrdB. Conversely, genes with a decreased expression in the presence of mercury included those coding for glutathione peroxidase and formamidase (Table 1). The expression of the *nitC* gene coding for the nitrilase NitC that is essential for cyanide assimilation was slightly decreased in the presence of mercury, although mercury had no effect on the expression of the *nitD* gene that is clustered together the *nitC* gene (Table 1).

**TABLE 1:** Transcriptional expression analysis by qRT-PCR of some *P. pseudoalcaligenes* CECT 5344 genes encoding proteins affected by mercury in the proteomic analysis

Gene^*a*^/protein ID^*b*^	Gene/protein name	Gene expression^*c*^ in:		Fold change^*d*^
		CN + Hg media	CN media	
BN5_3397/W6RJF8	PhoH family protein	0.156	0.086	1.82
BN5_4378/W6R2H6	Precorrin-2 C20-methyltransferase (CobI)	0.026	0.011	2.38
BN5_4162/W6R8H1	Alkanesulfonate monooxygenase (SsuD)	0.041	0.009	4.18
BN5_4165/W6RLE3	Antioxidant, AhpC/Tsa family (AhpC)	0.293	0.066	4.43
BN5_3403/W6QYN2	Thioredoxin reductase	0.418	0.083	5.00
BN5_2707/W6R4K3	ArsR family transcriptional regulator (ArsR2)	4.655	1.380	3.37
BN5_2708/W6QXT2	Arsenate reductase (ArsC3)	0.785	0.077	10.25
BN5_2412/W6R3L0	Glutathione *S*-transferase family protein	0.701	0.189	3.69
BN5_1290/W6R0E8	Ribonucleoside-diphosphate reductase (NrdB)	5.802	2.154	2.69
BN5_1595/W6R1A5	Glutathione peroxidase	0.528	0.650	0.81
BN5_3204/W6QY63	Formamidase (AmiF)	0.270	0.512	0.53
BN5_1632/H9N5E1	Nitrilase (NitC)	7.40	9.40	0.79
BN5_1633/H9N5E3	Radical SAM domain-containing protein (NitD)	5.524	5.536	1.00

a Protein code according to the Uniprot database under the accession number UP000032841.

b Gene annotation from GenBank (accession HG916826.1).

c Relative gene expression from cells grown in cyanide and mercury.

d Fold change represented as the ratio gene expression CN + Hg/CN.

### Transcriptional and bioinformatic analyses of MerR regulators.

The genome of *P. pseudoalcaligenes* CECT 5344 contains six genes that code for the following putative MerR-type transcriptional regulators: MerR1 (W6QQW2 protein/BN5_0701 gene), MerR2 (W6QVE0/BN5_2264), MerR3 (W6QWL9/BN5_2322), MerR4 (W6R6A1/BN5_3351), MerR5 (W6RKL7/BN5_3802), and MerR6 (W6R2T8/BN5_4473). BLASTP sequence comparisons of these six regulators revealed that MerR5 and MerR6 were homologs, displaying 50% identity. MerR2 showed 33% and 34% identity with MerR1 and MerR6, respectively, and MerR5 presented with 36.5% identity compared with MerR1 (see Table S2 in the supplemental material).

**FIG 4:**
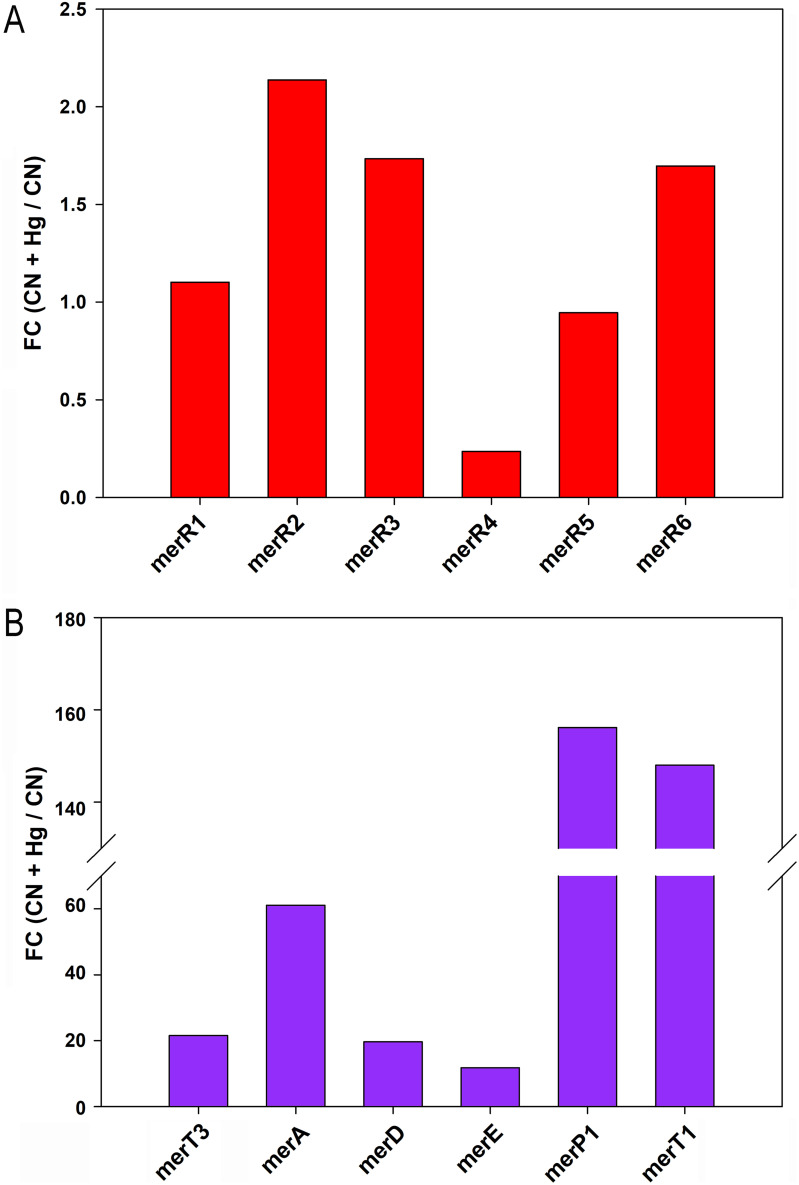
Transcriptional gene expression analysis by qRT-PCR of *P. pseudoalcaligenes* CECT 5344 *mer* genes. (A) Differential relative gene expression of *merR* genes. (B) Differential relative gene expression of other mercury-related genes. Gene expression was analyzed by qRT-PCR from cells grown with 2 mM sodium cyanide as the sole nitrogen source, without or with 75 μM HgCl_2_. The differential gene expression is represented as the fold change (FC) of the ratio relative gene expression (CN + Hg/CN). Gene/protein identifiers (IDs) are as follows: *merR1* (BN5_0701/W6QQW2), *merR2* (BN5_2264/W6QVE0), *merR3* (BN5_2322/W6QWL9), *merR4* (BN5_3351/W6R6A1), *merR5* (BN5_3802/W6RKL7) *merR6* (BN5_4473/W6R2T83), *merT3* (BN5_4474/W6R4B5), *merA* (BN5_4477/W6R9E5), *merD* (BN5_4478/W6R2U3), *merE* (BN5_4479/W6R4C0), *merP1* (BN5_3800/W6R0W8), and *merT1* (BN5_3801/W6R273).

A quantitative gene expression analysis of the *P. pseudoalcaligenes merR* genes was performed using mRNA from cells grown under cyanotrophic conditions in CN or CN + Hg media (Fig. 4A). Only *merR2* (BN5_2264) showed a significative induction by mercury, displaying a fold change of 2.2, whereas *merR4* (BN5_3351) was downregulated in the presence of mercury (Fig. 4A). Additionally, a transcriptional expression analysis of other genes that belong to the *merR6T3P3T5ADE* (BN5_4473 to BN5_4479) and *merP1T1R5* (BN5_3800 to BN5_3802) gene clusters of *P. pseudoalcaligenes* CECT 5344 was carried out by qRT-PCR. Genes included in these two *mer* operons of the CECT 5344 strain were highly induced in the presence of mercury. However, the transcriptional expression of the *merP1* and *merT1* genes located in the *merP1T1R5* gene cluster was higher than that displayed by genes of the *merR6T3P3T5ADE* gene cluster. In addition, the transcriptional expression of the mercuric (II) reductase *merA* gene (BN5_4477) was higher than the expression of the *merT3*, *merD*, and *merE* genes that are located in the same gene cluster (Fig. 4B).

**FIG 5:**
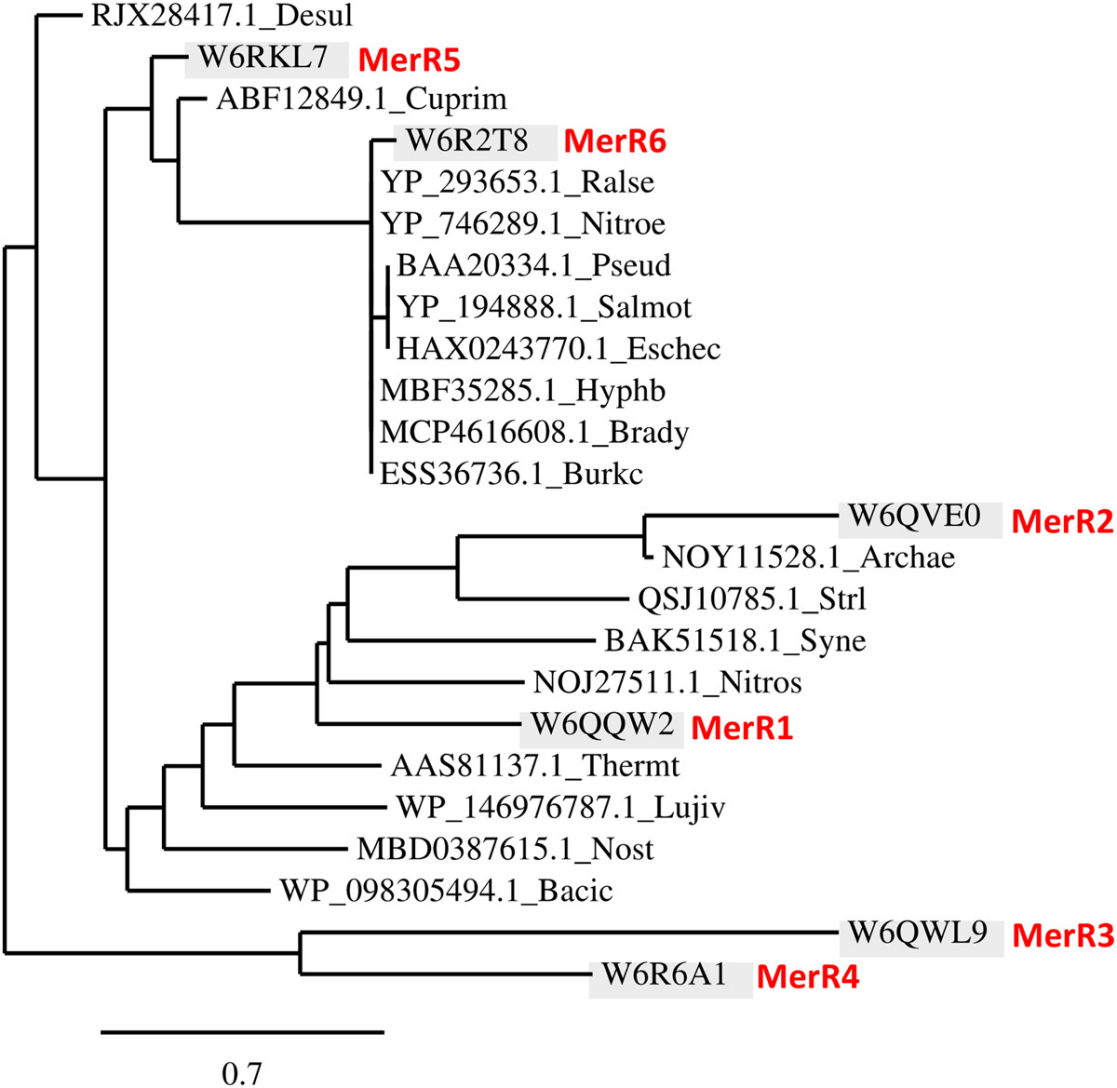
Phylogenetic distribution of MerR proteins in bacteria and archaea. The tree was constructed using the Phylogeny.fr platform (65). Sequences were aligned with MUSCLE v3.8.31 with default settings. Ambiguous regions were removed with Gblocks v0.91b. The phylogenetic tree was reconstructed using the maximum likelihood method implemented in the PhyML program 3.1/3.0 aLRT. The graphical representation and edition of the phylogenetic tree were performed with TreeDyn v198.3. The *P. pseudoalcaligenes* CECT 5344 MerR sequences are highlighted in red. Protein names (protein/gene IDs) are as follows: MerR1 (W6QQW2/BN5_0701), MerR2 (W6QVE0/BN5_2264), MerR3 (W6QWL9/BN5_2322), MerR4 (W6R6A1/BN5_3351), MerR5 (W6RKL7/BN5_3802), and MerR6 (W6R2T83/BN5_4473). Protein sequences correspond to the following phyla and organisms: A*lphaproteobacteria* (Hyphb, *Hyphomonadaceae bacterium*; Brady, *Bradyrhizobium* sp.), *Betaproteobacteria* (Burkc, Burkholderia cenocepacia KC-01; Nitroe, Nitrosomonas eutropha C91; Ralse, Ralstonia eutropha JMP134; Cuprim, Cupriavidus metallidurans CH34), Deltaproteobacteria (Desul, *Desulfarculus* sp.; Lujiv, *Lujinxingia vulgaris*), *Gammaproteobacteria* (Pseud, Pseudomonas sp. K-62), *Enterobacteria* (Salmot, Salmonella enterica subsp. *enterica* serovar Typhimurium; Eschec, Escherichia coli JJ1897), *Firmicutes* (Bacic, Bacillus cereus), *Actinobacteria* (Strl, Streptomyces lividans), *Cyanobacteria* (Syne, *Synechocystis* sp. PCC 6803; Nost, *Nostoc* sp. C3-bin3), *Deinococcus*-*Thermus* (Thermt, Thermus thermophilus HB27), and *Archaea* (Nitros, *Nitrososphaera* sp.; Archae, *Archaeoglobi archaeon*).

**FIG 6:**
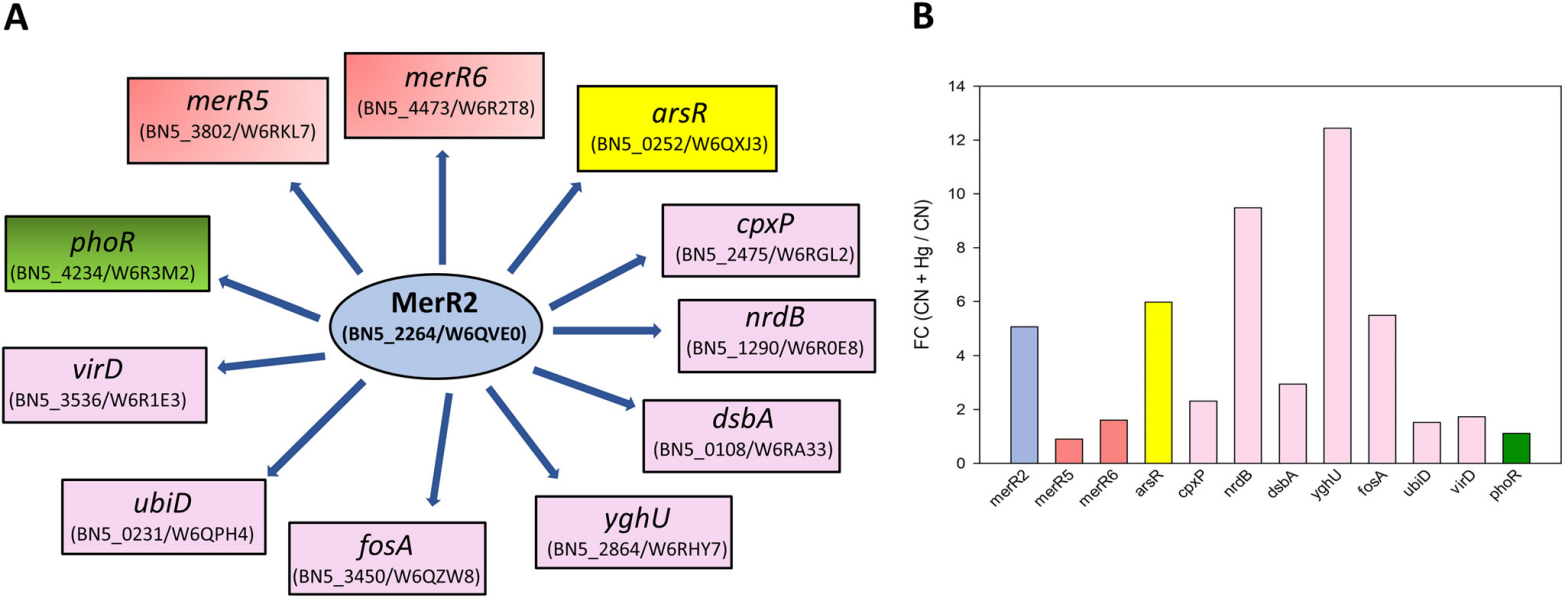
Hypothetical regulation network of the MerR2 regulon of *P. pseudoalcaligenes* CECT 5344 (A) and transcriptional qRT-PCR analysis of the MerR2-regulated genes (B). In this regulatory model, two MerR transcriptional regulator genes, namely, *merR5* and *merR6* (red boxes), are putatively under the control of the master regulator MerR2 (blue oval), as well as the transcriptional regulator *arsR* gene (yellow box) involved in arsenic resistance, the regulatory *phoR* gene that codes for the transcriptional regulator involved in phosphate metabolism (green box), and several structural genes (violet boxes), as follows: *cpxP*, chaperone involved in resistance to metals; *nrdB*, β-subunit of the ribonucleoside-diphosphate reductase; *dsbA*, dithiol oxidoreductase (disulfide-forming); *yghU*, glutathione *S*-transferase; *fosA*, glutathione *S*-transferase; *ubiD*, 3-octaprenyl-4-hydroxybenzoate carboxy-lyase; and *virD*, amino acid/peptide export protein. The transcriptional analysis by qRT-PCR shown in B was performed with mRNA from CECT 5344 cells harvested after 10 h of growth, when these genes showed the highest expression.

A bioinformatic analysis was performed to identify MerR orthologs in the KEGG database, revealing that MerR-type transcriptional regulators are distributed widely among proteobacteria and eubacteria, without significative differences in their distribution within the different phyla. Among the six MerR-type regulators of *P. pseudoalcaligenes* CECT 5344, MerR1 was the closest relative to MerR2 in the phylogenetic tree (Fig. 5). Curiously, the transcriptional regulator MerR2 was also very close, in evolutive distance, to those found in archaea and thermophilic bacteria (Fig. 5). An additional bioinformatic analysis was carried out to identify putative MerR2 binding boxes in the promoter gene regions of *P. pseudoalcaligenes* CECT 5344, according to the MerR binding sequence described previously in Escherichia coli (29). Thus, a predicted MerR2 binding sequence of 5′-(T/C)GTA(G/C)-N_4_-GTAC-3′ was identified in *P. pseudoalcaligenes* CECT 5344. This predicted MerR binding sequence was found in the promoters of several regulatory genes, including those involved in mercuric resistance (*merR5*, BN5_3802; and *merR6*, BN5_4473), in arsenic resistance (*arsR*, BN5_0252), and in phosphate metabolism (*phoR*, BN5_4234). It was also found in the promoters of structural genes for metal resistance (*cpxP*, BN5_2475), ribonucleoside-diphosphate reductase/β-subunit (*nrdB*, BN5_1290), dithiol oxidoreductase/disulfide-forming (*dsbA*, BN5_0108), two glutathione *S*-transferases (*yghU*, BN5_2864; and *fosA*, BN5_3450), 3-octaprenyl-4-hydroxybenzoate carboxy-lyase (*ubiD*, BN5_0231), and amino acid/peptide exporter (*virD*, BN5_3536) (Fig. 6A). Additionally, a transcriptomic study using qRT-PCR of the predicted MerR target genes was performed. In this analysis, six of the predicted target genes were significantly induced (FC >2) in the presence of mercury, including *arsR*, *cpxP*, *nrdB*, *dsbA*, *yghU*, and *fosA* genes. However, the expression of the genes *merR5*, *merR6*, and *phoR*, which code for regulatory proteins, was not significantly affected by mercury (Fig. 6B).

## DISCUSSION

### Mercury and cyanide resistance/detoxification by *P. pseudoalcaligenes* CECT 5344.

Mercury is considered one of most toxic elements that is present in the environment, threatening ecosystems and human health (2, 30). Mercury may form strong complexes with inorganic anions, such as sulfide, hydroxide, or chloride, and also with other metals like gold and silver (31, 32). In the environment, microorganisms can also produce organomercurial derivatives, mainly methylmercury (2, 33). Additionally, mercury can bind to cyanide, which is used at a large scale in mining activities through the cyanidation process (5). It has been proposed that cyanide might facilitate the methylation of mercury, thus increasing its solubility in anaerobic aqueous environments and the human body (34). Methylmercury is a highly toxic form of mercury that is also produced by anaerobic bacteria that harbor the *hgcAB* genes (35).

The method used most extensively in the laboratory for mercury removal from polluted wastewaters is based on ion-exchange resin adsorption, which is not suitable for environmental conditions. Bacterial-mediated detoxification of mercury is an advantageous technology because it is inexpensive, feasible, and allows the sequestration of mercury in diverse environments (2). Mercury-resistant bacteria usually contain *mer* genes that confer these microorganisms a great potential to be applied in mercury bioremediation techniques. These bacterial *mer* genes are relevant for the conversion of both reactive inorganic and organic forms of mercury to the volatile Hg^0^. Once in the atmosphere, Hg^0^ can be oxidized photochemically to Hg^II^, which is deposited back into terrestrial and aquatic ecosystems. Therefore, the occurrence of the Mer system in microorganisms can regulate the fate of mercury in the biogeochemical cycle of a specific environment (1, 36).

The cyanotrophic bacterium *P. pseudoalcaligenes* CECT 5344 uses free cyanides, metal cyanide complexes and cyano derivatives as the sole nitrogen source (24). In this bacterial strain, the cyanide degradation pathway occurs through the production of oxaloacetate, which reacts chemically with cyanide to generate a cyanohydrin that is converted into the corresponding carboxylic acid and ammonium by the nitrilase NitC. The ammonium released in the nitrilase reaction is incorporated into carbon skeletons through the glutamine synthetase/glutamate synthase cycle (8, 28). Sequencing of the whole genome of the strain CECT 5344 (25, 26) has allowed the development of different omic techniques applied to the degradation of cyanide present in different industrial wastes, including those generated from mining. Mercury and arsenic are usually present in these liquid residues that contain large amounts of cyanide, thus increasing their toxicity (7, 8). In this work, the simultaneous cyanide and mercury detoxifications by the cyanotrophic bacterium *P. pseudoalcaligenes* CECT 5344 have been characterized in minimal media with acetate and cyanide as the sole carbon and nitrogen source, respectively, in the presence or absence of mercuric chloride (Fig. 1). It is important to highlight that *P. pseudoalcaligenes* CECT 5344 showed higher MIC and MBC values for mercury in the presence of cyanide (200 μM and 300 μM, respectively) than with ammonium (10 μM and 17.5 μM, respectively), probably due to a lower toxicity of this metal when it forms complexes with cyanide. This result shows the advantage of using the strain CECT 5344 in the detoxification of industrial wastewaters containing both cyanide and mercury. Under cyanotrophic conditions, this bacterium tolerated elevated concentrations of mercury, displaying growth in the presence of 100 μM mercuric chloride. However, better growth and cyanide consumption rates were achieved in the presence of 75 μM mercuric chloride (Fig. 1). Additionally, the extracellular and intracellular determinations of mercury concentration by ICP-MS in the CECT 5344 cells grown with cyanide and mercury have revealed that this bacterial strain accumulated intracellularly a low amount of mercury, which is probably chelated to biomolecules. Likewise, most of the mercury added to the media was removed rapidly since mercury was not detected in the extracellular media after 7.5 h of growth, indicating that this bacterial strain detoxifies mercury efficiently in the presence of cyanide by transporting this metal inside the cell for its bioaccumulation/metabolization.

### Proteomic study of the resistance to mercury in *P. pseudoalcaligenes* CECT 5344 under cyanotrophic conditions.

To characterize at the molecular level the simultaneous detoxification by the strain CECT 5344 of these two potent toxics, namely, cyanide and mercury, and to determine the possible mechanisms involved in the mercury tolerance of this strain, a quantitative proteomic analysis was carried out. The proteomes from *P. pseudoalcaligenes* cells grown in the presence of 2 mM sodium cyanide as the sole nitrogen source, with 75 μM mercury chloride (CN + Hg) or without mercury (CN), were established (Fig. S1 to S4). A total of 2,689 proteins were identified in both proteomes, of which 209 proteins were differentially expressed and 21 were proteins found exclusively under the CN + Hg condition (Fig. S4; Table S1). Among the proteins identified in both proteomes, namely, CN and CN + Hg, were those encoded by the *nit1C* gene cluster, which codes for the nitrilase NitC and other proteins essential for cyanide assimilation in the strain CECT 5344 (28). Additionally, proteins encoded by the *cio* gene cluster were also identified in both proteomes (Fig. 2). These proteins include the cytochrome *bd*-type alternative oxidase CioAB that replaces the cytochrome *c* oxidase for a cyanide-independent respiration and the 4-hydroxy-tetrahydropicolinate synthase DapA1 and the nitrilase Nit4, which play a key role in intracellular iron homeostasis and 3-cyanoalanine assimilation, respectively (37, 38). Other members of the C-N hydrolase superfamily, including the nitrilases Nit1 and Nit2, were also detected in the proteomes from cells grown both in CN and CN + Hg media (Fig. 2). A mutational analysis of these nitrilase genes has revealed that they are not essential for cyanide degradation in the strain CECT 5344, but all together, they could contribute to the alleviation of cyanide toxicity, thus contributing to bacterial cyanide resistance (38). The fact that mercury did not significantly affect proteins involved in cyanide resistance and assimilation constitutes an advantage to apply *P. pseudoalcaligenes* CECT 5344 for cyanide and mercury bioremediation processes.

When the CN and CN + Hg proteomes were compared, the analysis of functional protein categories revealed that proteins overrepresented in the presence of mercury belonged to GO groups mainly related to the response to mercury ion, response to toxic substances, and detoxification of inorganic compounds (Fig. S5). Among proteins exclusive or overrepresented in the CN + Hg proteome were several proteins encoded by the two *mer* gene clusters of *P. pseudoalcaligenes* CECT 5344, including the MerR-family transcriptional regulator MerD, the mercuric reductase MerA, and the periplasmic mercury ion-binding proteins MerP1 and MerP3 (Fig. 3; Table S1). MerP and MerT proteins have been described to be essential to import mercury inside the cell. Mercury binds to two cysteine residues of the periplasmic protein MerP, being next transferred to the cytoplasmic protein MerT, which is also responsible for the uptake of organomercurials (39). MerT contains four cysteine residues by which reduced mercury is bound and transferred to the active side of the NADPH-dependent mercuric reductase MerA (23). The *merR6T3P3T5ADE* gene cluster (BN5_4473 to BN5_4479) of *P. pseudoalcaligenes* codes for additional proteins that were not identified in this proteomic study, including the mercuric resistance regulatory protein MerR6, the mercuric transport proteins MerT3 and MerT5, and the mercuric resistance protein MerE that functions as an inorganic/organic mercury transporter (40, 41). The second *merP1T1R5* gene cluster of the strain CECT 5344 (BN5_3800 to BN5_3802) also encodes two proteins that were not identified in this proteomic analysis, namely, the mercuric transport protein MerT1 and the mercuric resistance regulatory protein MerR5. It is worth noting that the detection of hydrophobic integral membrane proteins and regulators present in the cells at low concentrations are well-known limitations of proteomic techniques (42).

Mercury causes alterations in cell membrane permeability and DNA structure and provokes changes in the structure/function of proteins because it shows a high affinity to sulfhydryl groups (43). Glutathione (GSH) has a relevant role as an antioxidant, preventing the damage of essential cellular molecules in the presence of some metals or ion peroxides. Thus, mercury preferably binds to glutathione, cysteine, or homocysteine as a mercury resistance mechanism. GSH can also conjugate organomercurial compounds like methylmercury. In the proteomic study performed in this work, a glutathione *S*-transferase was found induced in the presence or mercury (Fig. 3; Table S1). This enzyme has a primary role in the conjugation of toxic substances to GSH and probably plays a crucial role in the cellular detoxification of mercury (43). Two GSH-dependent enzymes that were found downregulated in media with mercury, glutaredoxin, and glutathione peroxidase (Fig. 3; Table S1) are involved in redox reactions and protection from oxidative damage. This process is probably related to a decreased pool of biologically active GSH in the presence of mercury. In this sense, a thioesterase family protein was exclusive of the CN + Hg proteome (Fig. 3; Table S1). Thioesterases catalyze the cleavage of thioester groups present in a wide variety of molecules like coenzyme A, acyl carrier protein, and GSH, among others (44).

On the other hand, mercury induces oxidative stress by increasing reactive oxygen species, acting as a catalyst in Fenton-type reactions (45). Mercury also causes mitochondrial dysfunction, which can provoke alterations in calcium homeostasis and lipid peroxidation. In the proteomic analysis, the ATP-dependent hemin transporter HmuV was found exclusively in the CN + Hg proteome (Fig. 3; Table S1). Recently, it has been described that external hemin acts as an inhibitor of mitochondrial large-conductance calcium-activated potassium channel activity (46). Additionally, the antioxidant protein alkyl hydroperoxide reductase AhpC, which is a thiol-dependent enzyme, and the thiol-based redox sensor RegB were overrepresented in the CN + Hg proteome (Fig. 3; Table S1). Most redox signals are recognized by cysteine residues, which can be oxidized into different redox states like sulfenic, sulfinic, and sulfonic groups that can sense a range of oxidative signals. Thus, sulfenic acids can form complexes to GSH, and also two adjacent cysteine sulfenic acids can form intra- or intermolecular disulfide bonds, which allow redox sensor regulators to acquire specific conformations to modulate the expression of target genes (47). In this sense, components of an ABC-type sulfonate transporter, and also an alkanesulfonate monooxygenase that catalyzes the conversion of sulfonates into sulfite and the corresponding aldehyde, were found overrepresented in the CN + Hg proteome. Additionally, the sulfate-binding protein CysP3 was overrepresented in the presence of mercury (Fig. 3; Table S1). It has been described that Hg^II^ can react with sulfonates, and in fact, in human intoxication by mercury, a treatment based on oral administration of dimercaptopropane sulfonate, is recommended (43). It has also been described the role of the two-component regulatory RegAB system of Acidithiobacillus ferrooxidans in controlling ferrous iron and inorganic sulfur compounds oxidation (48).

Mercury may also react with pyrimidine nucleosides and nucleotides. In this sense, the enzymes dihydropyrimidine dehydrogenase and dihydropyrimidinase were overrepresented in the presence of mercury, as well as the uracil-xanthine permease and the α- and β-subunits of the ribonucleoside-diphosphate reductase (Fig. 3; Table S1). The ribonucleoside-diphosphate reductase catalyzes the reduction of ribonucleotides into their corresponding deoxyribonucleotides, and its activity relies on glutaredoxins and thiorredoxins. As mentioned above, glutaredoxin and thioredoxin were downregulated in the presence of mercury, as well as a thiol-disulfide isomerase/thioredoxin (Fig. 3; Table S1). Other proteins involved in DNA replication/repair and translation that were downregulated in the CN + Hg proteome included the DNA-binding protein HU, the TusC protein, the translation initiation factor IF-1, the ribosome-binding factor A, and the ribosome-recycling factor RRF (Table S1), suggesting that general cellular processes like DNA replication and translation are negatively affected by mercury.

Mercury also binds to several metallic cofactors present in enzymes that are essential for cell survival, leading to their downrepresentation in the CN + Hg proteome, such as several nonheme iron dioxygenases, different *c*-type cytochromes and several cytochrome *c*-containing proteins, the nickel/copper-containing superoxide dismutase, and the heme/Fe-S containing bacterioferritin (Fig. 3; Table S1). The formation of complexes between mercury (II) and the porphyrin ring, which is present in the heme group and cobalamin (vitamin B_12_), has been well described (49). In fact, a protein found exclusively in the presence of mercury was the cobalamin-independent methionine synthase, while the precorrin-2 C20-methyltransferase, which is involved in the synthesis of vitamin B_12_, was overrepresented in the presence of mercury, perhaps as a compensation mechanism. Curiously, different pyridoxal 5′-phosphate (PLP)-dependent enzymes were also found overrepresented in the CN + Hg proteome of the strain CECT 5344, such as cysteine desulfurase IscS, alanine racemase, β-alanine-pyruvate transaminase, and d-amino acid dehydrogenase (Fig. 3; Table S1). Cysteine desulfurase catalyzes the conversion of l-cysteine to l-alanine and sulfane sulfur through the formation of a protein-bound cysteine persulfide intermediate on a conserved cysteine. The proposed role of this enzyme is to participate in processes for the biosynthesis of Fe-S clusters, thiamine, tRNA thionucleosides, biotin, lipoic acid, molybdopterin, and NAD^+^ (50).

### Transcriptional and bioinformatic analysis of mercury detoxification in *P. pseudoalcaligenes* CECT 5344: an integrative view.

**FIG 7:**
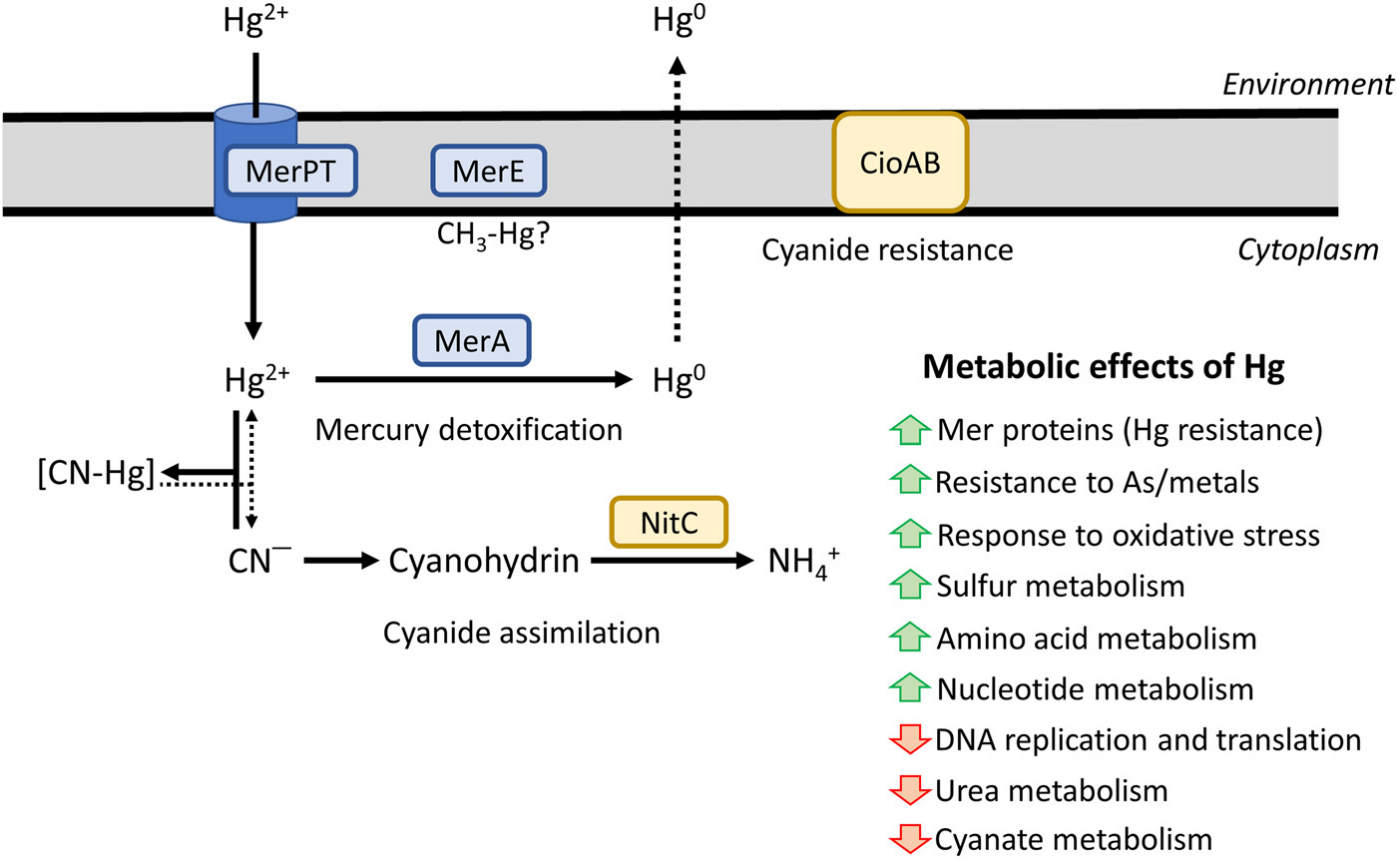
Overview of *P. pseudoalcaligenes* CECT 5344 metabolism under cyanotrophic conditions in the presence of mercury.

The expression of the *mer* genes depends on the activity of the regulator MerR, which acts as a transcriptional repressor in the absence of mercury. In the presence of reduced mercury, this metal binds to MerR, which undergoes an allosteric change that provokes its release from the promoter region, allowing active transcription of the structural *mer* genes. It has been proposed that the protein MerD may assist in the dissociation of MerR from DNA, probably by forming a complex with MerR, and therefore playing a coregulatory function in the expression of the *mer* genes (41). Although MerD was annotated in the genome of the strain CECT 5344 as a MerR-type transcriptional regulator, it is not homologous to the MerR regulators that are present in this bacterium. In addition to the mercuric regulatory proteins MerR6 and MerR5 encoded by the *mer* gene clusters from the CECT 5344 strain, four additional genes coding for putative MerR proteins (MerR1 to MerR4) were also identified in different *loci* of the genome. The transcriptional analysis of these six *merR* genes (*merR1* to *merR6*) encoding putative MerR transcriptional regulators in *P. pseudoalcaligenes* revealed that only the *merR2* gene was significatively induced by mercury when cyanide was the sole nitrogen source (Fig. 4A), suggesting that MerR2 may have a key role in the control of the *mer* genes. However, the expression of other *merR* genes slightly increased in the presence of mercury, and therefore, their role in the regulation of mercury resistance/detoxification in the strain CECT 5344 cannot be ruled out. The bioinformatic analysis of the distribution of MerR-type transcriptional regulators among bacteria and archaea showed that MerR2 has homologs in the primitively evolved archaea and thermophilic bacteria (Fig. 5), which indicates a very early origin of the mercury resistance mechanism in hydrothermal habitats that could contain elevated concentrations of mercury (2). Structural genes belonging to the *merR6T3P3T5ADE* and *merP1T1R5* gene clusters were highly induced in the presence of cyanide and mercury (Fig. 4B), thus indicating that probably MerR2 promotes in the presence of mercury the transcription of these *mer* genes that code for mercury transporters, mercury reductase, and the coregulator MerD. Thus, the strain CECT 5344 is a suitable candidate to be used in bioremediation techniques applied for the detoxification of industrial liquid wastes generated by the mining industry that usually contain elevated concentrations of cyanide and mercury, among other metals and metalloids like iron, copper, nickel, and arsenic (51). The results included in this work contribute to highlighting the great biodegradative potential of the CECT strain to detoxify highly toxic complex mixtures of contaminants (Fig. 7).

An additional bioinformatic analysis for searching MerR binding sites in the promoter regions of target genes of *P. pseudoalcaligenes* CECT 5344 allowed the identification of a putative MerR regulon that could control the mercury resistance/detoxification mechanisms in this bacterial strain, paralleling with cyanide detoxification/assimilation. The gene coding for MerR2 does not contain the predicted MerR binding box [5′-(T/C)GTA(G/C)-N_4_-GTAC-3′] in its promoter region. This result, together with the fact that *merR2* was the only *merR* gene of this bacterium that was significantly induced by mercury (Fig. 4A), lead us to propose that MerR2 could be a master regulator that controls the expression of different target genes that include the predicted MerR2 binding box in their promoter regions. Thus, different MerR2-regulated genes were induced in the presence of mercury, as described in the proteomic and/or transcriptional analyses carried out in this work (Fig. 3 and 6A; Table S1). Additionally, the *merR2* gene was identified previously as a putative target of a regulatory small RNA (sRNA258) that may control the detoxification of an industrial cyanide-containing residue by *P. pseudoalcaligenes* CECT 5344 (52). Integration of the proteomic data, transcriptional analysis, and bioinformatics has allowed us to predict a regulatory model for the MerR2 regulon of *P. pseudoalcaligenes* CECT 5344 that operates under cyanotrophic conditions (Fig. 6A). Among putative MerR2-regulated genes were *merR5* and *merR6*, which are located in the two *mer* gene clusters of this strain. Other putative MerR2-target genes were *cpxP*, which codes for a chaperone involved in the resistance to metals like mercury; the *nrdB* gene that codes for the β-subunit of the ribonucleoside-diphosphate reductase involved in DNA replication and repair, probably related to the damage that mercury causes directly to DNA; the *fosA* and *yghU* genes, which code for glutathione *S*-transferases that may contribute to increasing the GSH pool biologically active in the presence of mercury; and the dithiol oxidoreductase *dsbA gene*. Proteins of the Dsb system have been described to play a key role in the virulence of many pathogenic microorganisms by acting on the function of various protein secretion systems that influence the virulence (53). The bacterial strain CECT 5344 is not pathogenic but could be a mechanism of defense toward mercury toxicity. In this sense, another predicted target of MerR2 was the *virD* gene that codes for an amino acid/peptide export protein involved in quorum sensing. However, peptides and amino acids can be very effective, and often specific, ligands for a variety of metal ions like mercury. Additionally, the 3-octaprenyl-4-hydroxybenzoate carboxy-lyase *ubiD* gene involved in ubiquinone (coenzyme Q10) synthesis was also identified as a predicted MerR2 target. Recently, it has been shown that coenzyme Q10 supplementation alleviates the oxidative stress imposed by mercury in rats (54). On the other hand, the predicted MerR2-target *phoR* gene codes for a transcriptional regulator involved in phosphorus metabolism. Additionally, the phosphorus-related PhoH protein was found overrepresented in the presence of mercury in the proteomic study carried out in this work (Fig. 3; Table S1). PhoR is a transcriptional regulator that activates *pho* gene expression, while PhoH is a putative ATPase, without clear function. The Pho regulon in bacteria has been described to function not only as a simple regulatory circuit for controlling phosphate homeostasis but also as part of a complex regulatory network relevant for stress response (55). Phosphate is an essential nutrient for living organisms, including bacteria, and usually is scarce in the environment, thus limiting bacterial growth. Furthermore, mercury displays high affinity to phosphorus, making this nutrient even more limited in mercury-containing media. Finally, another predicted MerR2-target gene was *arsR* (BN5_0252), which codes for a transcriptional repressor that may control the expression of *ars* genes. Although arsenical compounds were not present in the culture media of the strain CECT 5344 in this study, several proteins encoded by the *P. pseudoalcaligenes* CECT 5344 *ars* genes involved in arsenic resistance were overrepresented in the CN + Hg proteome of this bacterium (Fig. 3; Table S1), including the thioredoxin-dependent arsenate reductase ArsC3, the ArsR2 transcriptional regulator, and the arsenic resistance protein ArsH2, which is an NADP^+^-dependent flavoprotein that protects against oxidative stress and confers resistance to organoarsenicals (56, 57). The presence of mercury in the growth media of *P. pseudoalcaligenes* leads to the induction of these proteins involved in arsenic resistance and detoxification, suggesting a possible cross talk between these two toxic elements, namely, mercury and arsenic. This result highlights the great biotechnological potential of the strain CECT 5344 for use in the detoxification of mining wastes that are composed of complex mixtures of highly toxic compounds like cyanide, mercury, and arsenic, which display different chemical properties that make their disposal very difficult.

## MATERIALS AND METHODS

### Culture media and bacterial growth conditions.

Pseudomonas pseudoalcaligenes CECT 5344 was cultured in M9 minimal media, at pH 9.5 and 30°C, under aerobic conditions with agitation of 220 rpm in an orbital shaker (24, 28). Sodium acetate (50 mM) was used as the carbon source, and sodium cyanide (2 mM) or ammonium chloride (2 mM) was used as the nitrogen source where indicated. When applicable, mercury chloride (Sigma-Aldrich) was added to the M9 media (from a 5 mM stock solution) to achieve the indicated concentration for each experiment. Agar plates were prepared by addition of 1.5% bacteriological agar to LB liquid medium.

### Determination of bacterial growth and mercury tolerance.

Bacterial growth was determined in liquid cultures by measuring the absorbance at 600 nm (A_600_) in a spectrophotometer (Spectronic; Thermo Fisher Scientific, USA). The tolerance of *P. pseudoalcaligenes* CECT 5344 to mercury was determined by calculation of the MIC and the minimum bactericidal concentration (MBC) (58). For this purpose, the strain CECT 5344 was grown (in quintuplicate) in U-shaped 96-well microtiter plates, containing M9 liquid medium, at 30°C with shaking at 220 rpm. After 48 h of incubation, the MIC of mercury that impairs visible growth of bacteria (MIC) and the minimum bactericide concentration of mercury that kills bacteria (MBC) were determined by measuring bacterial growth in agar plates by counting CFU with the drop plate technique (59).

### Analytical determinations.

Cyanide was determined colorimetrically in the presence of chloramine T, barbituric acid, and pyridine reagents as described previously (60). Protein was quantified by the method of Bradford (61).

The determination of mercury in the culture media or that accumulated extracellularly (biosorption) or intracellularly (chelated to biomolecules) was determined from 100-mL liquid cultures in M9 minimal media with 75 μM HgCl_2_ and 2 mM sodium cyanide, as the sole nitrogen source (CN + Hg media). Four biological samples were analyzed. Cells were centrifuged (10,000 rpm, 10 min, and 4°C) at 7.5 h of growth, and after centrifugation, supernatants were used to determine the mercury concentration. Pellets with cells were washed with 1 mL of a solution containing 0.85% NaCl. Cells were heated at 80°C for 96 h, and cell dry weight was determined. Pellets were digested in 69% HNO_3_ (trace-metal grade; Fisher). Mercury concentration was determined by inductively coupled plasma-mass-spectrometry (ICP-MS; PerkinElmer; model Nexion 350X) at the Central Service for Research Support of the University of Córdoba (SCAI-UCO).

### Quantitative proteomic analysis by LC-MS/MS.

Proteomic analysis was carried out from *P. pseudoalcaligenes* CECT 5344 cells grown in M9 minimal medium containing 50 mM sodium acetate and 2 mM sodium cyanide as the carbon and nitrogen sources, respectively, and in the presence of 75 μM HgCl_2_ (CN + Hg) or without mercury (CN). Four independent biological replicates were used for each medium. Cells were harvested by centrifugation at 12,000 rpm for 10 min at 4°C after 7.5 h of growth, when they were actively growing and most of the cyanide was depleted from the media. Pellets with cells were resuspended in 300 μL of a lysis buffer, containing 8 M urea, 50 mM Tris-HCl (pH 7.5), 4% 3-[(3-cholamidopropyl)-dimethylammonio]-1-propanesulfonate (CHAPS), and 1% SDS. Samples were disrupted by sonication in Bandelin Sonoplus H2070 equipment (8 pulses for 20 s, at 25 W). After centrifugation at 12,000 rpm for 10 min at 4°C, supernatants were cleaned by using the 2D-clean up kit (Amersham GE Healthcare). The LC-MS protocol, acquisition modes, data-independent acquisition-parallel accumulation serial fragmentation (DIA-PASEF) parameters, building up *in silico* library by using DIA-NN software, and searching criteria and quantification mode were carried out as described previously (62). The *P. pseudoalcaligenes* CECT 5344 proteome is available in Uniprot (UP000032841). Data were analyzed by using Perseus software (1.6.12.1). The principal-component analysis (PCA) was carried out, and a heat map and a volcano plot were generated by using default parameters (Fig. S1 to S3). Then a *t* test was applied, and differentially expressed proteins were defined as those with an adjusted *P* value of ≤0.05 and a log_2_ FC of ≥1 (overexpressed in CN + Hg media) or ≤−1 (overexpressed in CN media). Proteins indicated as exclusive were identified only under one condition (either CN + Hg or CN) in at least three of the four replicates from the same culture media. After data were filtered, a GO enrichment analysis was carried out by using the ComparativeGO application (27). Data were deposited to the ProteomeXchange Consortium (http://proteomecentral.proteomexchange.org) via the PRIDE partner repository with the data set identifier PXD038659.

### Quantitative real-time PCR analysis.

The transcriptional expression of selected genes was analyzed by qRT-PCR using three biological samples, each with two technical replicates. RNA isolation was performed following the Qiagen RNA extraction kit (RNeasy midi kit), and the synthesis of total cDNA was achieved with the SuperScript II reverse transcriptase (Invitrogen). The detailed procedure was described previously (63). Oligonucleotides sequences used as primers (see Table S3 in the supplemental material) in the qRT-PCRs were designed using the Oligo 7.0 software. The obtained data were normalized to the *dnaQ1* (BN5_2215) and the *dnaE* (BN5_2819) housekeeping genes. Gene expression was calculated by the threshold cycle ΔΔ*C_T_* method (62).

### Bioinformatic analysis of the MerR binding sequence in the genome of *P. pseudoalcaligenes*.

The pattern locator (PatLoc) software (https://academic.oup.com/bioinformatics/article/22/24/3099/210155) described previously (64) was used for the identification of MerR sequence patterns in the genome of *P. pseudoalcaligenes* CECT 5344 with the restriction of searching in intergenic regions. For that purpose, the sequence 5′-(T/C)GTA(G/C)-N_4_-GTAC-3′ was used as the predicted MerR binding box in *P. pseudoalcaligenes* CECT 5344, as deduced from the one described previously in E. coli (29).

### Statistical analysis.

Statistical significance was determined by a two-tailed *t* test analysis by using the Benjamini-Hochberg correction method. Data were analyzed by using Perseus software (v1.6.12.1) (https://maxquant.org/perseus/). Log_2_ LFQ intensity data were used for proteomics and normalized data for qRT-PCR. A pair of samples were considered significantly different when the adjusted *P* value was lower than 0.05. Perseus (v1.6.12.1) software was used for the proteomic data analysis. To identify the *merR* gene orthologs, the KEGG database was used (https://www.kegg.jp/kegg/kegg2.html). Other data were compared using the IBM SPSS Statistics v22 software.
