# Supplementary material for: Quantitative Proteomic Analysis of Cyanide and Mercury Detoxification by Pseudomonas pseudoalcaligenes CECT 5344
Source: Microbiol Spectr. 2023 Jul 11;11(4):e00553-23. doi: 10.1128/spectrum.00553-23 (PMC10433974; doi:10.1128/spectrum.00553-23)
Supplement: Supplemental file 1 — Supplemental material. Download spectrum.00553-23-s0001.docx, DOCX file, 1.2 MB [file spectrum.00553-23-s0001.docx]

**SUPPLEMENTAL MATERIAL**

**SUPPLEMENTARY FIGURE LEGENDS**

**FIG S1** Principal component analysis (PCA) of differential expression of proteins identified from *P. pseudoalcaligenes* CECT 5344 cells grown with cyanide as the sole nitrogen source either in media with 75 μM HgCl_2_ (CN + Hg) or without mercury (CN) media.

**FIG S2** Hierarchical cluster of differentially expressed proteins of *P. pseudoalcaligenes* CECT 5344 in the comparison of cells cultured with 2 mM sodium cyanide as the sole nitrogen source without mercury (CN) or with 75 μM HgCl_2_ (CN + Hg).

**FIG S3** Volcano plot of differentially expressed proteins of *P. pseudoalcaligenes* CECT 5344 in the comparison of cells cultured with 2 mM sodium cyanide as the sole nitrogen source without mercury (CN) or with 75 μM HgCl_2_ (CN + Hg).

**FIG S4** Venn diagram showing the number of proteins differentially expressed in *P. pseudoalcaligenes* CECT 5344 cells grown with 2 mM sodium cyanide as the sole nitrogen source without mercury (CN) or with 75 μM HgCl_2_ (CN + Hg).

**FIG S5** GO enrichment analysis among the proteins induced in the presence of 75 μM mercury. Enrichment is shown as % genes / GO term.

**
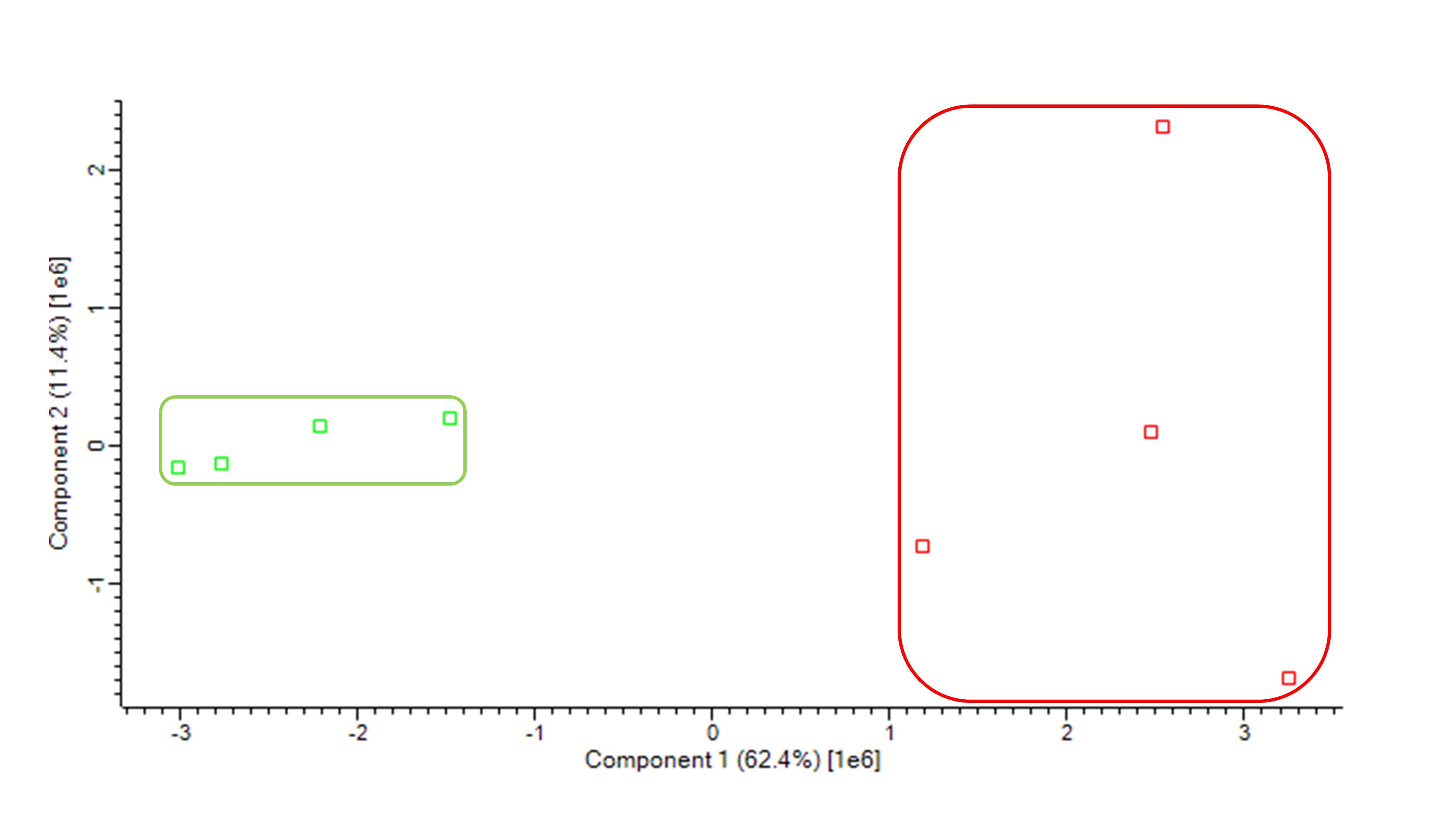
**

**FIGURE S1**


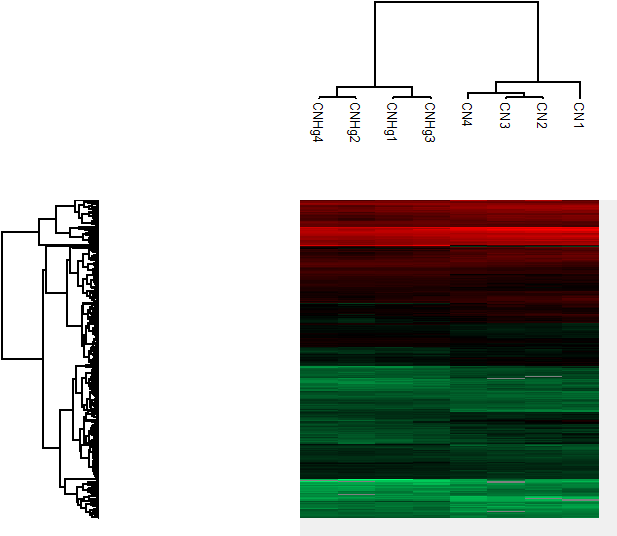


**FIGURE S2**


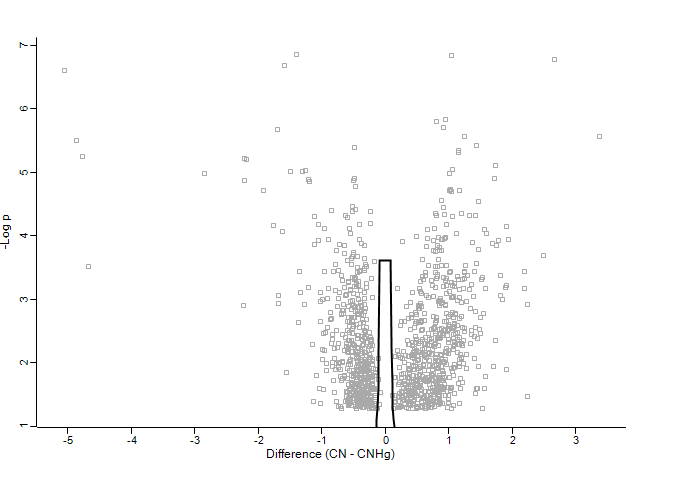


**FIGURE S3**

**
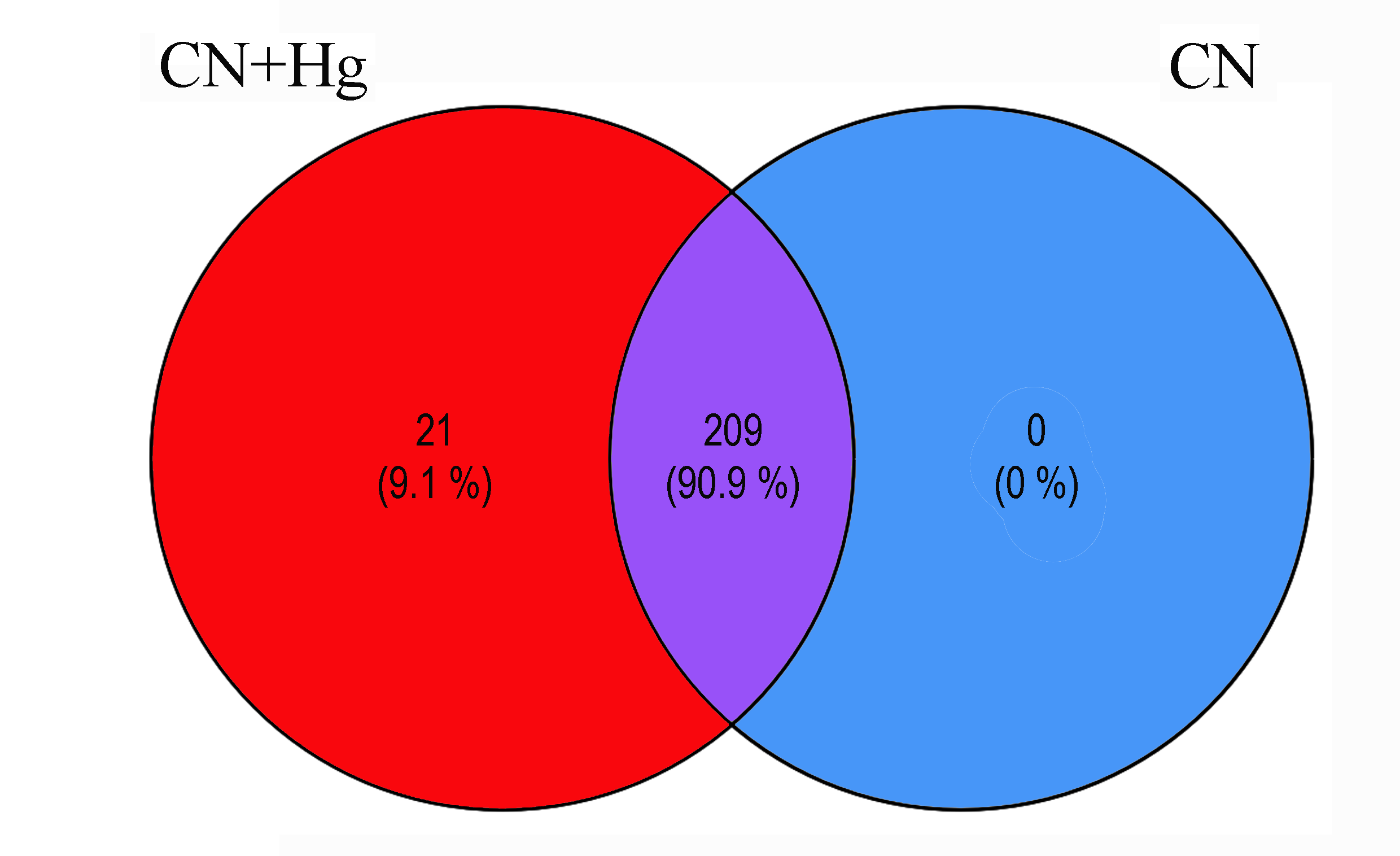
**

**FIGURE S4**

**
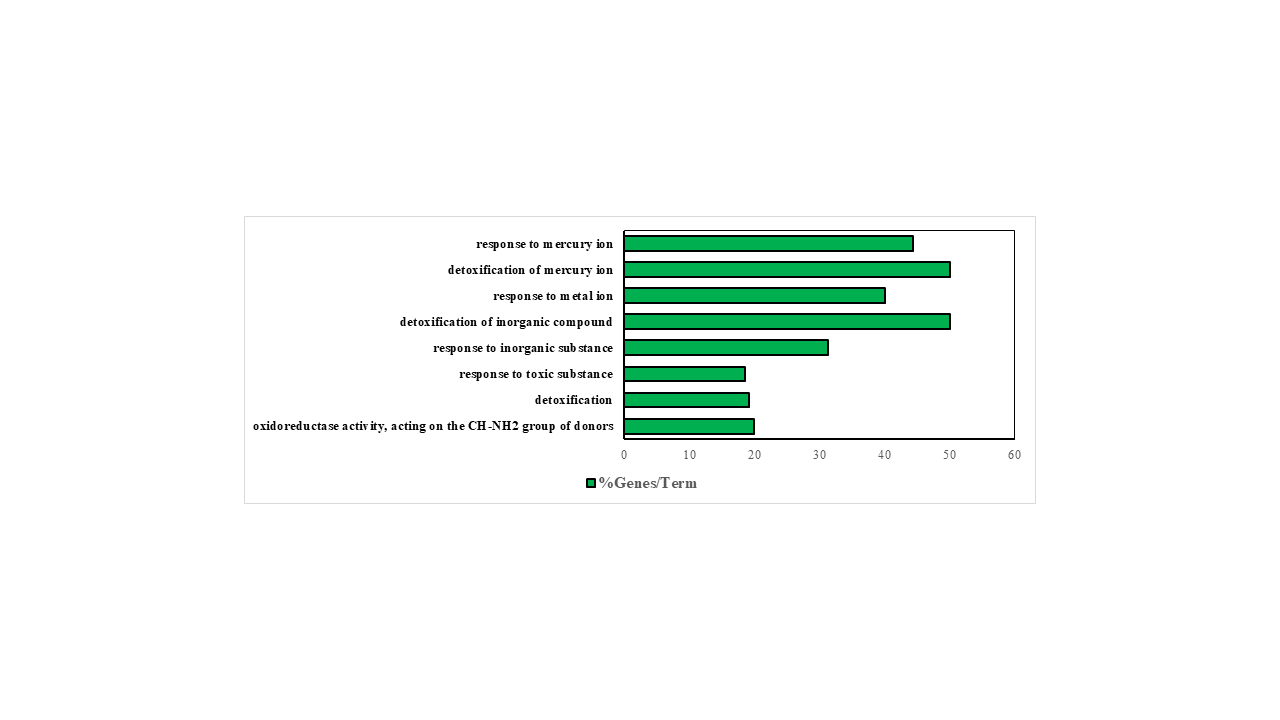
**

**FIGURE S5**

**SUPPLEMENTARY TABLES**

**TABLE S1** Effect of mercury on the proteome of *P. pseudocalcaligenes* cells grown under cyanotrophic conditions.

| **Protein ID^1^** | **Gene ID^2^** | **Name** | **Location** | **Log_2_ FC** | **Adjusted *p* value** |
| --- | --- | --- | --- | --- | --- |
| W6QQM8 | BN5_0198 | GCN5-related N-acetyltransferase (EC 2.3.1.57) | Cytoplasmic | Exclusive CN + Hg | - |
| W6QSS0 | BN5_0424 | Peptidase (EC 3.4.24.-) | Cytoplasmic | Exclusive CN + Hg | - |
| W6QRP5 | BN5_0560 | Uncharacterized protein | Unknown | Exclusive CN + Hg | - |
| W6QZG3 | BN5_0959 | Hemin import ATP-binding protein HmuV (EC 3.6.3.-) | Cytoplasmic/Membrane | Exclusive CN + Hg | - |
| W6QTI2 | BN5_1175 | Uncharacterized protein | Unknown | Exclusive CN + Hg | - |
| W6R0C1 | BN5_1260 | Uncharacterized protein | Unknown | Exclusive CN + Hg | - |
| W6QTV1 | BN5_1311 | Putative RuBisCO transcriptional regulator | Cytoplasmic | Exclusive CN + Hg | - |
| W6R1Z5 | BN5_1840 | NADH-quinone oxidoreductase subunit N (EC 1.6.5.11) | Cytoplasmic/Membrane | Exclusive CN + Hg | - |
| W6R283 | BN5_1927 | Putative 3-oxoadipate enol-lactonase (EC 3.1.1.1) | Cytoplasmic | Exclusive CN + Hg | - |
| W6R2G0 | BN5_2009 | Uncharacterized protein | Unknown | Exclusive CN + Hg | - |
| W6QVD5 | BN5_2259 | Thioesterase superfamily protein | Cytoplasmic | Exclusive CN + Hg | - |
| W6QY08 | BN5_2307 | AraC-family regulatory protein | Cytoplasmic | Exclusive CN + Hg | - |
| W6R3I0 | BN5_2382 | tRNA pseudouridine synthase A (EC 5.4.99.12) | Cytoplasmic | Exclusive CN + Hg | - |
| W6R403 | BN5_2546 | Uncharacterized protein | Unknown | Exclusive CN + Hg | - |
| W6RGU1 | BN5_2569 | Uncharacterized protein YqeB | Cytoplasmic | Exclusive CN + Hg | - |
| W6QZ36 | BN5_2694 | Cobalamin-independent methionine synthase (EC 2.1.1.14) | Cytoplasmic | Exclusive CN + Hg | - |
| W6QY66 | BN5_2862 | Uncharacterized protein | Unknown | Exclusive CN + Hg | - |
| W6RLD8 | BN5_4160 | Aliphatic sulfonates import ATP-binding protein SsuB (EC 3.6.3.-) (SsuB) | Cytoplasmic/Membrane | Exclusive CN + Hg | - |
| W6R0T9 | BN5_4161 | Putative aliphatic sulfonates transport permease protein (ssuC) | Cytoplasmic/Membrane | Exclusive CN + Hg | - |
| W6R108 | BN5_4201 | Chemotaxis response regulator protein-glutamate methylesterase 1 (EC 3.1.1.61) | Cytoplasmic | Exclusive CN + Hg | - |
| W6R2U3 | BN5_4478 | MerR family mercury resistance MerD protein | Unknown | Exclusive CN + Hg | - |
| W6RLE3 | BN5_4165 | Antioxidant, AhpC/Tsa family (EC 1.11.1.15) (AhpC3) | Cytoplasmic | 33.15 | 1.10E-05 |
| W6R9E5 | BN5_4477 | Mercuric-Hg(II) reductase (EC 1.16.1.1) MerA | Cytoplasmic | 29.15 | 2.10E-05 |
| W6RM71 | BN5_4475 | Periplasmic mercury ion-binding protein MerP3 | Cytoplasmic/Membrane | 27.19 | 3.43E-05 |
| W6R0W8 | BN5_3800 | Periplasmic mercury ion-binding protein MerP1 | Cytoplasmic/Membrane | 25.48 | 3.21E-03 |
| W6RLF8 | BN5_4180 | Nitrogen fixation protein AnfA | Cytoplasmic | 7.21 | 1.12E-05 |
| W6R8H1 | BN5_4162 | Alkanesulfonate monooxygenase (EC 1.14.14.5) (SsuB) | Cytoplasmic | 4.68 | 5.22E-03 |
| W6QWV0 | BN5_2413 | GntR family transcriptional regulator | Cytoplasmic | 4.63 | 1.20E-05 |
| W6QS35 | BN5_0164 | D-amino acid dehydrogenase (EC 1.4.99.-) | Cytoplasmic/Membrane | 4.63 | 3.12E-05 |
| W6RE84 | BN5_1603 | Glutamate dehydrogenase | Unknown | 4.56 | 3.20E-05 |
| W6QQZ8 | BN5_0328 | Dihydropyrimidine dehydrogenase (EC 1.3.1.2) | Cytoplasmic | 3.79 | 1.43E-05 |
| W6QPB8 | BN5_0166 | Alanine racemase (EC 5.1.1.1) | Cytoplasmic | 3.37 | 1.05E-03 |
| W6R3L0 | BN5_2412 | Glutathione *S*-transferase family protein (EC 2.5.1.18) | Cytoplasmic | 3.23 | 2.32E-05 |
| W6QS69 | BN5_1173 | Uncharacterized protein | Cytoplasmic/Membrane | 3.21 | 5.16E-03 |
| W6R2H6 | BN5_4378 | Precorrin-2 C20-methyltransferase (EC 2.1.1.130) | Cytoplasmic | 3.18 | 4.75E-03 |
| W6R1W0 | BN5_4183 | Sulfate-binding protein | Periplasmic | 3.05 | 9.28E-04 |
| W6QSH6 | BN5_0329 | Glutamate synthase (EC 1.4.1.13) | Cytoplasmic | 3.01 | 3.43E-05 |
| W6QY91 | BN5_2414 | MarR family transcriptional regulator | Unknown | 2.94 | 1.92E-02 |
| W6RAR6 | BN5_0330 | Dihydropyrimidinase (EC 3.5.2.2) | Cytoplasmic | 2.80 | 1.21E-05 |
| W6QZ50 | BN5_2709 | Arsenical resistance protein ArsH2 (EC 1.7.-.-) | Cytoplasmic | 2.64 | 3.20E-05 |
| W6R4K3 | BN5_2707 | Regulatory protein ArsR | Cytoplasmic | 2.58 | 6.80E-03 |
| W6RJI7 | BN5_3437 | Uracil-xanthine permease | Cytoplasmic/Membrane | 2.54 | 3.30E-03 |
| W6QZ30 | BN5_2689 | Indolepyruvate oxidoreductase subunit IorA (EC 1.2.7.8) | Cytoplasmic | 2.53 | 4.98E-03 |
| W6QRN5 | BN5_0983 | Uncharacterized protein | Cytoplasmic | 2.46 | 4.30E-05 |
| W6QSZ1 | BN5_1010 | Uncharacterized protein | Cytoplasmic | 2.41 | 5.08E-03 |
| W6QVZ9 | BN5_2100 | Uncharacterized protein | Cytoplasmic | 2.41 | 5.12E-03 |
| W6QYA4 | BN5_3249 | Uncharacterized protein | Cytoplasmic | 2.41 | 5.14E-03 |
| W6R107 | BN5_3391 | Uncharacterized protein | Cytoplasmic | 2.41 | 5.06E-03 |
| W6RJX0 | BN5_3552 | Uncharacterized protein | Cytoplasmic | 2.41 | 5.10E-03 |
| W6R1A0 | BN5_4291 | Uncharacterized protein | Cytoplasmic | 2.41 | 5.04E-03 |
| W6R8F5 | BN5_4147 | General secretion pathway protein E | Cytoplasmic | 2.40 | 4.32E-05 |
| W6QTT2 | BN5_1291 | Ribonucleoside-diphosphate reductase subunit alpha (EC 1.17.4.1) | Cytoplasmic | 2.32 | 3.40E-05 |
| W6QZ51 | BN5_0842 | Beta-alanine-pyruvate transaminase (EC 2.6.1.18) | Cytoplasmic | 2.30 | 4.60E-03 |
| W6R1N1 | BN5_1685 | UDP-N-acetylglucosamine 2-epimerase (EC 5.1.3.14) | Cytoplasmic | 2.27 | 2.00E-03 |
| W6QYN2 | BN5_3403 | Thioredoxin reductase (EC 1.8.1.9) | Unknown | 2.20 | 1.06E-02 |
| W6R911 | BN5_4332 | Uncharacterized protein | Unknown | 2.18 | 4.19E-02 |
| W6R0E8 | BN5_1290 | Ribonucleoside-diphosphate reductase subunit beta (EC 1.17.4.1) | Cytoplasmic | 2.17 | 2.64E-03 |
| W6RJ61 | BN5_3268 | Cysteine desulfurase IscS (EC 2.8.1.7) | Cytoplasmic | 2.15 | 1.19E-03 |
| W6R1R4 | BN5_1740 | Cytosine-specific methyltransferase (EC 2.1.1.37) | Cytoplasmic | 2.13 | 2.01E-02 |
| W6RJL0 | BN5_3457 | Cell division protein FtsZ | Cytoplasmic | 2.08 | 2.79E-03 |
| W6QZN2 | BN5_1039 | 2-Isopropylmalate synthase (EC 2.3.3.13) | Cytoplasmic | 2.07 | 1.10E-03 |
| W6QQY1 | BN5_0721 | Sensor histidine kinase RegB | Cytoplasmic/Membrane | 2.06 | 3.05E-02 |
| W6RKS1 | BN5_3902 | Urocanate hydratase (Urocanase) (EC 4.2.1.49) | Cytoplasmic | 2.03 | 4.95E-03 |
| W6QXT2 | BN5_2708 | Arsenate reductase ArsC3 (thioredoxin-dependent) (EC 3.1.3.48) | Cytoplasmic | 2.03 | 6.77E-03 |
| W6RJF8 | BN5_3397 | PhoH family protein | Cytoplasmic | 2.02 | 5.18E-03 |
| W6RAD4 | BN5_0165 | Endoribonuclease L-PSP | Cytoplasmic | 2.02 | 4.33E-02 |
| W6R1F7 | BN5_4356 | Antitoxin | Unknown | -2.00 | 1.27E-02 |
| W6QYX7 | BN5_2663 | Plasmid stabilization protein | Unknown | -2.00 | 6.68E-03 |
| W6RI94 | BN5_2964 | Isochorismatase hydrolase (EC 3.5.1.19) | Cytoplasmic | -2.00 | 3.00E-02 |
| W6QYG3 | BN5_0579 | Urease subunit beta (EC 3.5.1.5) (Urea amidohydrolase subunit beta) | Cytoplasmic | -2.00 | 6.42E-03 |
| W6R236 | BN5_3765 | Uncharacterized protein | Unknown | -2.01 | 8.81E-03 |
| W6QRC4 | BN5_0866 | Uncharacterized protein | Unknown | -2.01 | 3.60E-02 |
| W6QSX2 | BN5_1431 | Uncharacterized protein | Unknown | -2.02 | 2.21E-02 |
| W6QQH3 | BN5_0593 | ABC-type branched-chain amino acid transport systems periplasmic component-like protein | Unknown | -2.02 | 1.73E-03 |
| W6RAA1 | BN5_0139 | ABC transporter, solute-binding component | Periplasmic | -2.03 | 3.51E-03 |
| W6QSM0 | BN5_0374 | Substrate-binding region of ABC-type glycine betaine transport system | Unknown | -2.03 | 4.55E-03 |
| W6QU53 | BN5_1854 | Uncharacterized protein | Unknown | -2.03 | 3.43E-05 |
| W6QWP5 | BN5_2353 | Uncharacterized protein | Unknown | -2.03 | 6.84E-03 |
| W6QTK0 | BN5_1200 | Uncharacterized protein | Unknown | -2.03 | 3.16E-02 |
| W6RAK9 | BN5_0260 | Putrescine-binding periplasmic protein | Periplasmic | -2.03 | 3.37E-03 |
| W6QZ82 | BN5_3191 | Pyrimidine biosynthesis enzyme THI13 | Unknown | -2.03 | 1.88E-03 |
| W6QS83 | BN5_0229 | Thioredoxin | Cytoplasmic | -2.04 | 6.32E-03 |
| W6QR81 | BN5_0408 | Putative polyhydroxyalkanoic acid system protein | Cytoplasmic | -2.05 | 3.05E-02 |
| W6QV86 | BN5_1302 | 2-Dehydro-3-deoxyphosphogluconate aldolase (EC 4.1.3.16) | Cytoplasmic | -2.05 | 1.12E-06 |
| W6QQT7 | BN5_0671 | Antitoxin | Unknown | -2.05 | 4.90E-02 |
| W6QPR3 | BN5_0321 | UPF0312 protein BN5_0321 | Unknown | -2.05 | 4.11E-03 |
| W6RAQ0 | BN5_0310 | Response regulator receiver protein | Cytoplasmic | -2.05 | 1.79E-02 |
| W6RH38 | BN5_2664 | Protein ParD | Unknown | -2.06 | 2.97E-02 |
| H9N5E1 | BN5_1632 | Nitrilase NitC1 (EC 3.5.5.7) | Cytoplasmic | -2.06 | 1.68E-03 |
| W6QY63 | BN5_3204 | Formamidase (EC 3.5.1.49) (Formamide amidohydrolase) | Cytoplasmic | -2.07 | 5.00E-03 |
| W6QT84 | BN5_0581 | Urease subunit gamma (EC 3.5.1.5) (Urea amidohydrolase subunit gamma) | Cytoplasmic | -2.07 | 2.91E-02 |
| W6R5H8 | BN5_3069 | Uncharacterized protein | Unknown | -2.08 | 1.88E-02 |
| W6R7R9 | BN5_3899 | Endonuclease I (EC 3.1.21.1) | Extracellular | -2.08 | 4.82E-03 |
| W6QVD3 | BN5_1347 | Uncharacterized ABC transporter extracellular-binding protein PH1214 | Periplasmic | -2.09 | 4.98E-03 |
| W6QXA7 | BN5_2940 | OmpA/MotB domain-containing protein | Cytoplasmic/Membrane | -2.09 | 1.16E-03 |
| W6QQL3 | BN5_0178 | Nitrogen regulatory protein P-II | Cytoplasmic/Membrane | -2.09 | 6.93E-03 |
| W6R1B7 | BN5_3511 | SSU ribosomal protein S30P / sigma 54 modulation protein | Cytoplasmic | -2.09 | 5.82E-03 |
| W6QY43 | BN5_2354 | ABC transporter periplasmic protein | Unknown | -2.09 | 3.12E-06 |
| W6RK65 | BN5_3642 | N utilization substance protein B homolog (Protein NusB) | Cytoplasmic | -2.10 | 8.72E-03 |
| W6R2X7 | BN5_2155 | Uncharacterized protein | Unknown | -2.10 | 4.47E-02 |
| W6QWV2 | BN5_0020 | Type IV pilus assembly PilZ | Unknown | -2.10 | 8.39E-03 |
| W6R0I3 | BN5_3670 | 50S ribosomal protein L17 | Cytoplasmic | -2.11 | 2.01E-02 |
| W6QRT7 | BN5_0605 | Acetyl-CoA carboxylase biotin carboxyl carrier protein subunit (EC 6.4.1.2) | Cytoplasmic | -2.11 | 1.13E-02 |
| W6QU90 | BN5_1901 | Uncharacterized protein | Unknown | -2.11 | 1.20E-02 |
| W6RFG7 | BN5_2062 | Putative ring-cleaving dioxygenase | Unknown | -2.11 | 1.20E-02 |
| W6QWG1 | BN5_2660 | Flagellar hook-associated protein 2 (HAP2) (Flagellar cap protein) | Extracellular | -2.11 | 1.76E-02 |
| W6QXY6 | BN5_2282 | Peptidase | Unknown | -2.11 | 4.58E-02 |
| W6QX82 | BN5_2905 | Sigma factor AlgU regulatory protein MucB | Periplasmic | -2.11 | 4.71E-03 |
| W6R248 | BN5_1897 | Thiol-disulfide isomerase and thioredoxin protein | Unknown | -2.11 | 7.02E-03 |
| W6QTN7 | BN5_1245 | Soluble aldose sugar dehydrogenase yliI (EC 1.1.5.-) | Cytoplasmic/Membrane | -2.12 | 6.32E-03 |
| W6QTY0 | BN5_1774 | Methyl-accepting chemotaxis protein tlpB | Cytoplasmic/Membrane | -2.12 | 6.72E-03 |
| W6QYA3 | BN5_2424 | Orotidine 5'-phosphate decarboxylase (EC 4.1.1.23) (OMP decarboxylase) (OMPDCase) (OMPdecase) | Cytoplasmic | -2.12 | 1.64E-02 |
| W6R293 | BN5_3821 | Transcriptional regulator, putative | Cytoplasmic | -2.13 | 1.80E-02 |
| W6R0J6 | BN5_4079 | Membrane-bound lytic murein transglycosylase F | Periplasmic | -2.13 | 6.66E-03 |
| W6REZ4 | BN5_1868 | Translation initiation factor IF-1 | Cytoplasmic | -2.13 | 1.05E-02 |
| W6R242 | BN5_4228 | Uncharacterized protein | Unknown | -2.13 | 4.74E-03 |
| W6QWV6 | BN5_1882 | Protein tusC | Unknown | -2.13 | 3.42E-02 |
| W6R0A0 | BN5_1229 | ABC transporter, binding protein | Unknown | -2.13 | 6.70E-03 |
| W6QQD5 | BN5_0553 | Urease accessory protein UreF | Unknown | -2.13 | 4.47E-03 |
| W6QX98 | BN5_0125 | GlcG protein | Unknown | -2.13 | 9.99E-03 |
| W6QXY4 | BN5_2772 | Uncharacterized protein | Unknown | -2.13 | 9.61E-03 |
| W6QZU3 | BN5_1094 | Lipoprotein, putative | Unknown | -2.16 | 7.59E-03 |
| W6QS10 | BN5_0673 | Nucleotidyltransferase substrate binding protein | Unknown | -2.16 | 6.44E-03 |
| W6QYD7 | BN5_0554 | Urease accessory protein UreE | Cytoplasmic | -2.16 | 6.34E-03 |
| W6QPL1 | BN5_0266 | Putrescine transport system substrate-binding protein | Periplasmic | -2.17 | 2.44E-03 |
| W6RKA7 | BN5_3677 | 50S ribosomal protein L15 | Cytoplasmic | -2.19 | 5.22E-03 |
| W6QQ36 | BN5_0441 | ABC transporter/ATPase component protein | Cytoplasmic/Membrane | -2.20 | 3.77E-03 |
| W6R2G4 | BN5_3896 | NADPH:quinone oxidoreductase 1 (EC 1.6.5.2) | Cytoplasmic | -2.20 | 6.76E-03 |
| W6R4U4 | BN5_2831 | Ribosome-recycling factor (RRF) (Ribosome-releasing factor) | Cytoplasmic | -2.20 | 7.49E-03 |
| W6R3S3 | BN5_2472 | YciI like Protein | Unknown | -2.20 | 8.83E-03 |
| W6QZU8 | BN5_2968 | Cold shock protein (Beta-ribbon, CspA family) | Cytoplasmic | -2.21 | 1.25E-02 |
| W6R8K1 | BN5_4192 | Uncharacterized protein | Unknown | -2.21 | 2.30E-06 |
| W6RIL1 | BN5_3062 | Peptidyl-prolyl cis-trans isomerase (PPIase) (EC 5.2.1.8) | Periplasmic | -2.22 | 4.05E-03 |
| W6QS79 | BN5_1188 | Uncharacterized protein | Unknown | -2.22 | 2.58E-02 |
| H9N5E3 | BN5_1633 | Radical SAM domain-containing protein (Radical SAM domain-containing proteinBiotin synthase-related enzymeRibosomal RNA large subunit methyltransferase N) (EC 2.1.1.-) | Unknown | -2.23 | 1.23E-05 |
| W6QWQ0 | BN5_2358 | Uncharacterized protein | Unknown | -2.24 | 1.62E-02 |
| W6R2J3 | BN5_2039 | Uncharacterized protein | Unknown | -2.24 | 4.37E-03 |
| H9N5D9 | BN5_1636 | Uncharacterized protein | Unknown | -2.24 | 1.78E-03 |
| W6R0V6 | BN5_3784 | 30S ribosomal protein S21 | Cytoplasmic | -2.25 | 6.24E-03 |
| W6R9E0 | BN5_4472 | Uncharacterized protein | Unknown | -2.25 | 4.33E-02 |
| W6QXE9 | BN5_2096 | Uncharacterized protein | Unknown | -2.26 | 4.84E-03 |
| W6QWZ6 | BN5_0060 | Uncharacterized protein | Unknown | -2.26 | 1.70E-02 |
| W6QYQ7 | BN5_0634 | Antitoxin | Unknown | -2.26 | 1.50E-02 |
| W6RC99 | BN5_0905 | UPF0125 protein BN5_0905 | Unknown | -2.26 | 1.05E-02 |
| W6R1G2 | BN5_4361 | Uncharacterized protein | Unknown | -2.26 | 8.67E-03 |
| W6QWH2 | BN5_1752 | DNA-binding protein HU | Cytoplasmic | -2.27 | 1.28E-02 |
| W6R3W1 | BN5_2507 | Uncharacterized protein | Unknown | -2.27 | 3.05E-02 |
| W6QQN7 | BN5_0208 | Peptidyl-prolyl cis-trans isomerase (EC 5.2.1.8) | Unknown | -2.28 | 2.23E-02 |
| W6QU93 | BN5_0931 | Ribosome-binding factor A | Cytoplasmic | -2.29 | 1.40E-02 |
| W6RLY4 | BN5_4365 | 31 kDa Immunogenic protein | Unknown | -2.29 | 5.09E-03 |
| W6QQQ9 | BN5_0638 | Uncharacterized protein | Cytoplasmic | -2.29 | 1.03E-02 |
| W6QZI3 | BN5_3307 | 3-Oxoacid CoA-transferase (EC 2.8.3.5) | Cytoplasmic | -2.30 | 1.40E-02 |
| W6QQ87 | BN5_0036 | Substrate-binding region of ABC-type glycine betaine transport system | Unknown | -2.30 | 1.33E-03 |
| W6QV53 | BN5_1267 | Uncharacterized protein | Periplasmic | -2.30 | 1.38E-02 |
| W6REZ7 | BN5_1873 | Outer-membrane lipoprotein carrier protein | Periplasmic | -2.30 | 6.46E-03 |
| W6QZG6 | BN5_3683 | 30S ribosomal protein S14 | Cytoplasmic | -2.32 | 4.07E-03 |
| W6RIM2 | BN5_3078 | TPR repeat-containing protein | Unknown | -2.32 | 1.08E-02 |
| W6R0J9 | BN5_3685 | 50S ribosomal protein L24 | Cytoplasmic | -2.33 | 7.01E-03 |
| W6R1Q1 | BN5_4471 | Uncharacterized protein | Unknown | -2.33 | 2.54E-02 |
| W6R131 | BN5_4211 | Keratin, type II cytoskeletal 75 | Unknown | -2.34 | 9.36E-03 |
| W6QWE3 | BN5_2236 | Uncharacterized protein | Unknown | -2.35 | 1.62E-02 |
| W6QYJ4 | BN5_2987 | Branched-chain amino acid transport system substrate-binding protein | Periplasmic | -2.35 | 4.53E-03 |
| W6R4Q8 | BN5_2791 | Uncharacterized protein | Unknown | -2.37 | 7.43E-03 |
| W6QTU1 | BN5_1301 | 6-Phosphogluconolactonase (EC 3.1.1.31) | Unknown | -2.37 | 1.20E-06 |
| W6QZI8 | BN5_3312 | YceI family protein | Unknown | -2.38 | 3.58E-03 |
| W6QU34 | BN5_1391 | Uncharacterized protein | Unknown | -2.38 | 3.39E-03 |
| W6R648 | BN5_3281 | Uncharacterized protein | Cytoplasmic | -2.39 | 7.00E-03 |
| W6R0L4 | BN5_3207 | Basic membrane protein A2 Immunodominant antigen P39 | Unknown | -2.39 | 3.53E-03 |
| W6QXC5 | BN5_0151 | Flavin monoamine oxidase-related protein (EC 1.4.3.4) | Unknown | -2.41 | 3.29E-03 |
| W6QVQ2 | BN5_2381 | N-(5'-phosphoribosyl)-anthranilate isomerase (PRAI) (EC 5.3.1.24) | Cytoplasmic | -2.42 | 1.28E-02 |
| W6R3F4 | BN5_2347 | Cytochrome c550 | Unknown | -2.44 | 5.13E-03 |
| W6RHL4 | BN5_2751 | Ornithine utilization regulator | Cytoplasmic | -2.44 | 1.81E-02 |
| W6QXS7 | BN5_3119 | Putative quercetin 2,3-dioxygenase PA3240 (EC 1.13.11.24) | Cytoplasmic | -2.46 | 3.46E-03 |
| W6R265 | BN5_1912 | Nitrilase Nit4 (EC 3.5.5.1) | Cytoplasmic | -2.51 | 1.23E-03 |
| W6RKF0 | BN5_3692 | 30S ribosomal protein S19 | Cytoplasmic | -2.52 | 4.39E-03 |
| W6R1A7 | BN5_3501 | Toluene tolerance family protein | Unknown | -2.55 | 5.57E-03 |
| W6R0H7 | BN5_1325 | Uncharacterized protein | Unknown | -2.55 | 1.03E-02 |
| W6RJ65 | BN5_3273 | Uncharacterized protein | Unknown | -2.55 | 4.40E-03 |
| W6RAS2 | BN5_0335 | Cytochrome c | Unknown | -2.56 | 8.03E-03 |
| W6R1Y7 | BN5_1830 | Acetyltransferase, GNAT family | Cytoplasmic | -2.57 | 7.28E-03 |
| W6QZS8 | BN5_1079 | Thiol:disulfide interchange protein DsbC (EC 5.3.4.1) | Periplasmic | -2.60 | 2.73E-03 |
| W6R0G7 | BN5_4048 | Uncharacterized protein | Unknown | -2.62 | 4.67E-03 |
| W6RCV7 | BN5_1112 | Bacterioferritin (EC 1.16.3.1) | Cytoplasmic | -2.64 | 3.49E-03 |
| W6R2Y2 | BN5_2160 | Arylesterase (EC 3.1.2.-) | Unknown | -2.64 | 8.61E-03 |
| W6QYR8 | BN5_0649 | Type IV pilus assembly PilZ | Unknown | -2.65 | 1.40E-02 |
| W6RGQ5 | BN5_2520 | Rhodanese domain-containing protein | Cytoplasmic | -2.67 | 5.70E-03 |
| W6RGB7 | BN5_2355 | Beta-propeller repeat protein | Unknown | -2.68 | 1.21E-03 |
| W6QYF6 | BN5_2952 | Outer membrane porin | Outer Membrane | -2.68 | 7.04E-03 |
| W6QXB9 | BN5_2950 | Uncharacterized protein | Unknown | -2.68 | 3.43E-02 |
| W6QQB2 | BN5_0076 | Extracellular solute-binding protein | Periplasmic | -2.70 | 3.49E-03 |
| W6R924 | BN5_4347 | Uncharacterized protein | Unknown | -2.71 | 3.04E-02 |
| W6QST4 | BN5_0439 | ABC-type transporter periplasmic component protein | Cytoplasmic/Membrane | -2.71 | 1.32E-05 |
| W6REJ6 | BN5_1683 | Integration host factor subunit beta (IHF-beta) | Cytoplasmic | -2.73 | 3.03E-02 |
| W6RGC1 | BN5_2360 | Uncharacterized protein | Unknown | -2.73 | 8.70E-03 |
| W6QZC1 | BN5_3648 | Uncharacterized protein | Unknown | -2.76 | 1.70E-02 |
| W6R1F1 | BN5_4010 | Spermidine/putrescine-binding periplasmic protein 1 | Periplasmic | -2.76 | 1.60E-03 |
| W6R0K4 | BN5_1355 | Uncharacterized protein | Cytoplasmic | -2.77 | 3.60E-03 |
| W6QY35 | BN5_0462 | Uncharacterized protein | Unknown | -2.79 | 7.40E-03 |
| W6QZH6 | BN5_2848 | Cold shock protein (Beta-ribbon, CspA family) | Cytoplasmic | -2.82 | 7.57E-03 |
| W6QSF0 | BN5_0823 | Uncharacterized protein | Unknown | -2.83 | 6.28E-03 |
| W6R5G1 | BN5_3059 | Uncharacterized protein | Unknown | -2.86 | 4.00E-03 |
| W6QYY5 | BN5_3131 | Secreted protein-like protein | Unknown | -2.88 | 3.72E-03 |
| W6R347 | BN5_2205 | Hydroxysteroid dehydrogenase-like protein 2 | Unknown | -2.88 | 4.90E-02 |
| W6R1A5 | BN5_1595 | Glutathione peroxidase | Periplasmic | -2.92 | 6.36E-03 |
| W6RCU6 | BN5_1102 | Uncharacterized protein | Outer Membrane | -2.96 | 3.05E-02 |
| W6RM46 | BN5_4445 | Glutaredoxin | Unknown | -2.97 | 9.41E-04 |
| W6QW31 | BN5_1612 | Uncharacterized protein | Unknown | -2.99 | 2.12E-02 |
| W6QSK2 | BN5_0354 | Uncharacterized protein | Unknown | -3.00 | 4.42E-03 |
| W6RCP9 | BN5_1062 | UPF0135 protein | Unknown | -3.02 | 1.60E-03 |
| W6RDM6 | BN5_1378 | Uncharacterized protein | Unknown | -3.22 | 2.67E-03 |
| W6R217 | BN5_1865 | Cold shock-like protein CspG | Cytoplasmic | -3.26 | 1.71E-02 |
| W6RAC3 | BN5_0149 | Putative endoribonuclease L-PSP | Unknown | -3.29 | 1.21E-06 |
| W6R0U1 | BN5_3308 | Butyryl-CoA:acetate CoA transferase (EC 2.8.3.5) | Cytoplasmic | -3.32 | 2.32E-05 |
| W6RFI0 | BN5_2077 | Uncharacterized protein | Unknown | -3.34 | 9.18E-03 |
| W6QUE9 | BN5_1006 | Putative amino-acid ABC transporter-binding protein | Periplasmic | -3.37 | 3.04E-03 |
| W6QY14 | BN5_0442 | Cyanate hydratase (Cyanase) (EC 4.2.1.104) (Cyanate hydrolase) (Cyanate lyase) | Cytoplasmic | -3.43 | 2.82E-03 |
| W6QRT0 | BN5_0092 | Cytochrome c oxidase subunit 2 (EC 1.9.3.1) | Cytoplasmic/Membrane | -3.50 | 3.44E-03 |
| W6R2R0 | BN5_2094 | Uncharacterized protein | Unknown | -3.50 | 4.77E-03 |
| W6R7X3 | BN5_3959 | Uncharacterized protein | Unknown | -3.59 | 5.35E-03 |
| W6RIH7 | BN5_3015 | Superoxide dismutase [Cu-Zn] (EC 1.15.1.1) | Periplasmic | -3.71 | 4.65E-03 |
| W6R2G2 | BN5_4363 | Uncharacterized protein | Unknown | -3.75 | 4.57E-03 |
| W6RB18 | BN5_0440 | ABC transporter inner membrane subunit protein | Cytoplasmic/Membrane | -3.75 | 1.03E-03 |
| W6QWV3 | BN5_2780 | Periplasmic nitrate reductase, electron transfer subunit (Diheme cytochrome c NapB) | Periplasmic | -3.77 | 1.84E-02 |
| W6RLM7 | BN5_4230 | Integration host factor subunit alpha | Cytoplasmic | -3.84 | 2.93E-03 |
| W6QPA6 | BN5_0150 | Cytochrome C6 | Unknown | -4.57 | 4.44E-03 |
| W6RGF7 | BN5_2405 | Blue (Type1) copper domain-containing protein | Unknown | -4.58 | 3.44E-03 |
| W6QZ04 | BN5_3156 | Uncharacterized protein | Unknown | -4.74 | 4.96E-03 |
| W6R0B4 | BN5_3998 | Uncharacterized protein | Unknown | -4.74 | 3.65E-02 |
| W6QXU7 | BN5_2242 | Rhodanese domain-containing protein | Unknown | -5.64 | 3.30E-03 |
| W6QQ99 | BN5_0056 | TRAP dicarboxylate transporter-DctP subunit | Unknown | -6.32 | 2.31E-05 |
| W6QSQ4 | BN5_0409 | Poly(Hydroxyalkanoate) granule-associated protein (Phasin) | Cytoplasmic | -10.39 | 1.23E-05 |

*P. pseudoalcaligenes* CECT 5344 cells were grown with 2 mM cyanide as sole nitrogen source, either in the presence of 75 μM mercury (CN + Hg) or in the absence of mercury (CN). (1) Protein code according to Uniprot database under the accession number UP000032841. (2) Genes annotation from GeneBank (Accession HG916826.1). (3) Log_2_ fold change (FC), represented as the ratio normalized peptide intensity in CN + Hg media / normalized peptide intensity in CN media. After the t-test analysis was applied, the differential expressed proteins showed a p-value ≤ 0.05 (Benjamini-Hochberg corrected). Exclusive CN + Hg: proteins that were only detected in cells grown in CN + Hg media.

**TABLE S2** Sequence comparisons of the *P. pseudoalcaligenes* CECT 5344 MerR transcriptional regulators.

| **% Identity** | **MerR1** | **MerR2** | **MerR3** | **MerR4** | **MerR5** | **MerR6** |
| --- | --- | --- | --- | --- | --- | --- |
| **MerR1** | 100.0 | 33.3 | - | - | 36.5 | 28.9 |
| **MerR2** |  | 100.0 | - | - | 29.9 | 34.3 |
| **MerR3** |  |  | 100.0 | 24.4 | 28.0 | - |
| **MerR4** |  |  |  | 100.0 | 29.5 | 26.5 |
| **MerR5** |  |  |  |  | 100.0 | 50.0 |
| **MerR6** |  |  |  |  |  | 100.0 |

(-) No significative identity. Name (protein/gene IDs): MerR1 (W6QQW2/BN5_0701), MerR2 (W6QVE0/BN5_2264), MerR3 (W6QWL9/BN5_2322), MerR4 (W6RW6A1/BN5_3351), MerR5 (W6RKL7/BN5_3802) and MerR6 (W6R2T8/BN5_4473).

**TABLE S3** Oligonucleotides used in the qRT-PCR analysis of *P. pseudocalcaligenes* CECT 5344.

| **Primer name^*^** | **Name** | **Sequence (5’-3’)** |
| --- | --- | --- |
| BN5_0108qRTF | Thiol:disulfide interchange protein | CGATCGCGAAGCCTTCCTCAAGACCT |
| BN5_0108qRTR |  | ACGATACTTGCCGCCGACGACCA |
| BN5_0231qRTF | 3-octaprenyl-4-hydroxybenzoate carboxy-lyase | ACGCCAGTGCCCGATACCCTC |
| BN5_0231qRTR |  | CGCCCGGATGGATCACACCCT |
| BN5_0252qRTF | ArsR family transcriptional regulator | CCTGATCGCCTGTTGCTGCTCT |
| BN5_0252qRTR |  | ATACCGAGCAGCGCCTCCAG |
| BN5_0701qRTF | MerR family transcriptional regulator | AGTCCATAGATCTGCTGCCCCATGCC |
| BN5_0701qRTR |  | TGCCGACTTCCTCCAGCGAAAAGCC |
| BN5_1290qRTF | Ribonucleoside-diphosphate reductase beta chain | ACCAACCCCGAGTGCCGCCAGT |
| BN5_1290qRTR |  | CGCCTTCATCCATGCCCAGCGATT |
| BN5_1595qRTF | Glutathione peroxidase | TCCGGTGCCAATGCCATCCCGTTG |
| BN5_1595qRTR |  | ACTCGCCACCACCTTGCCCTG |
| BN5_1632qRTF | Nitrilase NitC | TGAGCGTATGGTCTGGGGGCAGGG |
| BN5_1632qRTR |  | CGCCGCATGAATCTGCTCGCCATC |
| BN5_1633qRTF | Radical SAM domain-containing protein NitD | CCGTGGTGCTGCCATCCTTTGCGAAT |
| BN5_1633qRTR |  | CAAGCGGCGAAACCAGGCGTCCTC |
| BN5_2215qRTF | DNA polymerase III, epsilon subunit | CCCCAACAACTCGACACAACCG |
| BN5_2215qRTR |  | GAGAAGCAAGGCGTGACAACCG |
| BN5_2264qRTF | MerR family transcriptional regulator | CTCGACATCACCACCCGCGCCAT |
| BN5_2264qRTR |  | CGCGCAGGATGAGTTTCAGCGTCACC |
| BN5_2322qRTF | MerR family transcriptional regulator | ACTCTGCGCGGTCAAGCCCCAC |
| BN5_2322qRTR |  | ACCCCGCCGCTTTACCGGAT |
| BN5_2412qRTF | Glutathione *S*-transferase | AGCGAGTTTCTGCCGACCGAGCC |
| BN5_2412qRTR |  | AGAGCTGGATGAAACGCGCCACC |
| BN5_2475qRTF | Periplasmic protein CpxP/Spy | AAGACCCTCACCGCCCTGCT |
| BN5_2475qRTR |  | TCCTGGCGCTGCTCCTTGCT |
| BN5_2707qRTF | ArsR family transcriptional regulator | ATGCCATGTCGCCAACCGAAGTCT |
| BN5_2707qRTR |  | CGCCTCTTCCAGTGCCGAGGTCA |
| BN5_2708qRTF | Arsenate reductase | TTTTCTTTGGCCCGGCGACTCGTT |
| BN5_2708qRTR |  | CGCTCACTCGCTTCTGGATCTGCT |
| BN5_2819qRTF | DNA-directed DNA polymerase | TCCCCCTTCAATGCCTCACCG |
| BN5_2819qRTR |  | CCTGCGTCTGCGTGCCAAGCG |
| BN5_2864qRTF | Putative glutathione *S*-transferase like protein | CGCCGAACAACAAGATTCCCGCCAT |
| BN5_2864qRTR |  | CAGAATTGCCCCGGACTCGAACAGC |
| BN5_3204qRTF | Formamidase | ACCGCACCGATCCGTCACTCGT |
| BN5_3204qRTR |  | ACACACTGGCCGTCTGCATCAGGT |
| BN5_3351qRTF | MerR family transcriptional regulator | CACCGGCCAATCTCGCACGCAT |
| BN5_3351qRTR |  | TGCTTGCGGCACCTGCTCATGGTC |
| BN5_3397qRTF | PhoH family protein | CGCTACCGCAGGATCATCGCCACC |
| BN5_3397qRTR |  | GCTCTCGTCCTCCATGTGCAACGCTT |
| BN5_3403qRTF | Thioredoxin reductase | CGCCAACCTGAAACCGCTGCTGA |
| BN5_3403qRTR |  | GTCACCCGGCCAGTTGTCCACCT |
| BN5_3450qRTF | Glutathione *S*-transferase domain-containing protein | CTACCCCGAGGCGCAGCTGT |
| BN5_3450qRTR |  | CGCCAGATCCATCGGCAGGTGAC |
| BN5_3536qRTF | Type IV secretion system protein VirD4 | ATCGAGCGCCGCCACAAGCTAC |
| BN5_3536qRTR |  | GCCAGCGCCGTCTCGAAGAAGTCCA |
| BN5_3800qRTF | Periplasmic mercury ion-binding protein | CCGTGCTTTTCGCCTTGCCCTT |
| BN5_3800qRTR |  | GCCGGACACCTTCTCCAGCGACT |
| BN5_3801qRTF | Mercuric transport protein | TGGCATTCCGCAAGCTCTACCTGGTG |
| BN5_3801qRTR |  | AGCACGCTGACGATCCAGAACACGAG |
| BN5_3802qRTF | Mercury regulatory protein | AGTCGTGCGCCTGTGCCGAA |
| BN5_3802qRTR |  | ATCGTTCCGCCGTCTCCCGCAT |
| BN5_4162qRTF | Alkanesulfonate monooxygenase | CCAAGCTGCTCTACCCGCCGAT |
| BN5_4162qRTR |  | CGCCCCAGGTCAGGTACAGCTC |
| BN5_4165qRTF | Antioxidant, AhpC/Tsa family | ACCGGGCGCAACTTCAATGAAATCCT |
| BN5_4165qRTR |  | CAACGACGGCACGATCACCACCT |
| BN5_4234qRTF | Signal transduction histidine kinase | GGCCTGAAAACCCCGCAAGACAGC |
| BN5_4234qRTR |  | AGTTGCCACGCTCGAAGTAGTCCT |
| BN5_4378qRTF | Precorrin-2 C20-methyltransferase | CTCGCCCCGCCGCTGAGCTAC |
| BN5_4378qRTR |  | CGCAGATCACCGCCACGTCCT |
| BN5_4473qRTF | Mercuric resistance operon regulatory protein | GCACCCATTGCGAGGAAGCCAGCAG |
| BN5_4473qRTR |  | ACCAAATCAGACAGCACGGCCTCCAT |
| BN5_4474qRTF | Mercuric transport protein | CGGGTGAGGTTTGCGCGATTCCCCAA |
| BN5_4474qRTR |  | CCGAGCGCGACCAAAACCAGCAC |
| BN5_4477qRTF | Mercuric reductase (Hg(II) reductase) | CTCGCCATCCGCAACCGCAT |
| BN5_4477qRTR |  | CGCCGCAAGCTTCAACCCCT |
| BN5_4478qRTF | MerD protein | ACTACCTGCTGCGCGGATTGCT |
| BN5_4478qRTR |  | CTCGAAGGCCGCCCGCACGAA |
| BN5_4479qRTF | Putative mercuric resistance protein MerE | ACCTGTCCCTGCCATTTGCCGAT |
| BN5_4479qRTR |  | ATCTTTGAAGGCCCGCAGCAGT |
